# Remodeling of the tumor microenvironment via disrupting Blimp1^+^ effector Treg activity augments response to anti-PD-1 blockade

**DOI:** 10.1186/s12943-021-01450-3

**Published:** 2021-11-20

**Authors:** Michael L. Dixon, Lin Luo, Sadashib Ghosh, Jeffrey M. Grimes, Jonathan D. Leavenworth, Jianmei W. Leavenworth

**Affiliations:** 1 Department of Neurosurgery, University of Alabama at Birmingham, 1600 6th Avenue South, CHB 118A, Birmingham, AL 35233 USA; 2 Graduate Biomedical Sciences Program, University of Alabama at Birmingham, Birmingham, AL 35294 USA; 3 School of Pharmacy, Nantong University, Nantong, Jiangsu 226001 China; 4 The O’Neal Comprehensive Cancer Center, University of Alabama at Birmingham, Birmingham, AL 35294 USA; 5 Department of Dermatology, University of Alabama at Birmingham, Birmingham, AL 35294 USA; 6 Department of Microbiology, University of Alabama at Birmingham, Birmingham, AL 35294 USA

**Keywords:** Anti-tumor immunity, Humoral antibody response, IgE, Effector regulatory T-cells, Follicular regulatory T-cells, Treg lineage stability, Tumor remodeling, Response to checkpoint blockade

## Abstract

**Background:**

Accumulation of Foxp3^+^ regulatory T (Treg) cells in the tumor often represents an important mechanism for cancer immune evasion and a critical barrier to anti-tumor immunity and immunotherapy. Many tumor-infiltrating Treg cells display an activated phenotype and express the transcription factor Blimp1. However, the specific impact of these Blimp1^+^ Treg cells and their follicular regulatory T (T_FR_) cell subset on tumor and the underlying mechanisms of action are not yet well-explored.

**Methods:**

Various transplantable tumor models were established in immunocompetent wild-type mice and mice with a Foxp3-specific ablation of Blimp1. Tumor specimens from patients with metastatic melanoma and TCGA datasets were analyzed to support the potential role of Treg and T_FR_ cells in tumor immunity. In vitro culture assays and in vivo adoptive transfer assays were used to understand how Treg, T_FR_ cells and antibody responses influence tumor control. RNA sequencing and NanoString analysis were performed to reveal the transcriptome of tumor-infiltrating Treg cells and tumor cells, respectively. Finally, the therapeutic effects of anti-PD-1 treatment combined with the disruption of Blimp1^+^ Treg activity were evaluated.

**Results:**

Blimp1^+^ Treg and T_FR_ cells were enriched in the tumors, and higher tumoral T_FR_ signatures indicated increased risk of melanoma metastasis. Deletion of Blimp1 in Treg cells resulted in impaired suppressive activity and a reprogramming into effector T-cells, which were largely restricted to the tumor-infiltrating Treg population. This destabilization combined with increased anti-tumor effector cellular responses, follicular helper T-cell expansion, enhanced tumoral IgE deposition and activation of macrophages secondary to dysregulated T_FR_ cells, remodeled the tumor microenvironment and delayed tumor growth. The increased tumor immunogenicity with MHC upregulation improved response to anti-PD-1 blockade. Mechanistically, Blimp1 enforced intratumoral Treg cells with a unique transcriptional program dependent on Eomesodermin (Eomes) expression; deletion of Eomes in Blimp1-deficient Treg cells restored tumor growth and attenuated anti-tumor immunity.

**Conclusions:**

These findings revealed Blimp1 as a new critical regulator of tumor-infiltrating Treg cells and a potential target for modulating Treg activity to treat cancer. Our study has also revealed two *FCERIA*-containing immune signatures as promising diagnostic or prognostic markers for melanoma patients.

**Supplementary Information:**

The online version contains supplementary material available at 10.1186/s12943-021-01450-3.

## Background

The immune responses and self-tolerance are stringently controlled by Foxp3^+^ Treg cells, but accumulation of these Treg cells within the tumor represents a major obstacle to the development of effective anti-tumor immunity and immunotherapy [1–5]. The frequency of Foxp3^+^ Treg cells among tumor-infiltrating lymphocytes (TIL) is often associated with poor prognosis of patients with various cancers [1–5]. According to phenotypic and functional specialization, Foxp3^+^ Treg cells are categorized into central Treg and effector Treg (eTreg) subsets [6, 7]. The eTreg subset displays an activated phenotype and effector program, and expresses the transcription factor (TF) Blimp1 (encoded by *Prdm1*) [7, 8]. TIL Blimp1-expressing Treg cells have been recently proposed to be included for outcome prediction of some cancer patients [9]. We and others have also established that Blimp1 is required for the lineage stability and suppressive activity of Foxp3^+^ Treg cells during ongoing immune or inflammatory responses [10–13]. However, the contribution of Blimp1^+^ Treg cells to tumor progression remains largely unclear. Their tumor-specific regulatory activities and the impact of the tumor microenvironment (TME) on their function are entirely unknown.

Our recent study has also revealed that Blimp1 is required for the stability and suppressive activity of T_FR_ cells that belong to a type of eTreg cells. Expression of Blimp1 in T_FR_ cells ensures the proper regulation of follicular helper T (T_FH_) cells, B-cells and germinal center (GC) antibody (Ab) responses [10, 13]. While increased TIL T_FH_ cells and B-cells as well as the formation of tertiary lymphoid structures (TLS) are associated with favorable outcomes in patients with certain types of cancer and better responses to checkpoint blockade therapies [14–18], the contribution of T_FR_ cells and humoral Ab responses to the regulation of anti-tumor immunity remains poorly understood.

Here, using various transplantable tumor models, we evaluated how the TME imprints Blimp1^+^ Treg cells and how disruptions of their suppressive activity reshape local and systemic immune responses as well as responses to PD-1 checkpoint blockade.

## Methods

All reagents or resources are listed in Additional File 1, if not specified in the text.

### Mice and human samples

C57BL/6 J (B6), *Prdm1*^fl/fl^, *Foxp3*^YFP-Cre^, *Rosa26*^Cre-ERT2^, *Eomes*^fl/fl^, *Tcrα*^−/−^ and *Prdm1*^EYFP^ (Blimp1-YFP) (Jackson Labs) mice were housed in pathogen-free conditions. *Prdm1*^fl/fl^ mice were bred onto *Foxp3*^YFP-Cre^ or *Rosa26*^Cre-ERT2^ mice to generate *Prdm1*^fl/fl^*Foxp3*^YFP-Cre^, *Prdm1*^fl/+^*Foxp3*^YFP-Cre^ or *Prdm1*^fl/fl^*Rosa26*^Cre-ERT2^ mice, respectively. *Prdm1*^fl/fl^*Foxp3*^YFP-Cre^ mice were further crossed onto *Eomes*^fl/fl^ mice to yield *Eomes*^fl/fl^*Prdm1*^fl/fl^*Foxp3*^YFP-Cre^ (double knockout, DKO) mice. All mice were used at the age of 5 to 10 weeks unless otherwise specified. Both sexes (males or females) were randomly included for comparison groups in all experiments in an unblinded fashion. Generally, 3-7 mice were used per group unless otherwise indicated in each experiment. De-identified tissue samples from patients with stage IV metastatic melanoma and control tissues were provided from the University of Alabama at Birmingham (UAB) Tissue Collection and Banking Facility. The characteristic of these samples is listed in Additional file 2.

### Cell lines

B16-F10 melanoma cells were purchased from American Type Culture Collection. B16-F10, B16 cells expressing the surrogate antigen ovalbumin (B16-OVA) or B16 expressing granulocyte-macrophage colony stimulating factor, GM-CSF (B16-GVAX) and MC38 colon cancer cells were cultured in complete Dulbecco’s Modified Eagle Medium (DMEM; Millipore Sigma) containing 10% FBS (Atlanta Biologicals) and 1× Penicillin/Streptomycin (Millipore Sigma), as described previously [19, 20]. 250 μg/mL of G418 was added into the B16-OVA tumor cell line culture. All tumor cell lines were pathogen free, used within three to eight passages, and maintained at 37 °C with 5% CO_2_.

### Tumor models

Mice were implanted subcutaneously (s.c.) with 2.5 × 10^5^ B16-F10 or MC38 cells, or 2.5–4 × 10^5^ B16-OVA cells on the flank on day 0. Mice implanted with B16-OVA cells were intraperitoneally (i.p.) immunized with NP-OVA in complete Freund’s adjuvant (CFA) on day 0 and NP-OVA in incomplete Freund’s adjuvant (IFA) on day 7. In some cases, mice implanted with B16-OVA cells were immunized s.c. with 1 × 10^6^ irradiated (150 Gy) GVAX on the opposite flank on day 1, and then 1 × 10^6^ irradiated B16-OVA and GVAX on alternating flanks on days 3 and 7. Tumor volume was measured 2–3 times per week using calipers and calculated as (x × y × z)/2 mm^3^. For mice treated with anti-PD-1, 200 μg anti-PD-1 or rat IgG2a isotype control (BioXcell) was i.p. injected into mice at days 3,6,9 post-tumor inoculation, according to the protocol established by others [21–23]. Mice with the tumor reaching 2 cm on the longest axis or with > 10% ulcerated tumor or with < 2 body condition score, according to the UAB Animal Care and Use Committee (IACUC) guidelines, were euthanized before the end of the study. Mice were euthanized by CO_2_ inhalation followed by cervical dislocation.

### Cell isolation

The spleen was extracted, and a single cell suspension was obtained by mashing the spleen between frosted microscope slides. Red blood cells were then removed using the Ammonium-Chloride-Potassium (ACK) lysis buffer and the cell suspension was filtered through a 70 μm filter membrane to eliminate debris. To isolate single cells from B16 melanoma or fresh human tissues, tumors or control tissues were mechanically disassociated into small pieces (< 3 mm) followed by an agitated digestion for 1 h at 37 °C in a dissociation solution (PBS supplemented with 2% FBS, 1 mg/ml collagenase/Dispase and 0.5 mg/ml DNase I for B16 or PBS supplemented with 2% FBS, 0.5 mg/ml collagenase/Dispase for human tissues). Digested samples were washed with DMEM/2% FBS and passed through a 70 μm cell strainer, and then separated on a Ficoll-Paque 1.084 density gradient (40% mixed with cells/80% for B16, or 75%/100% with the cell solution layered on the top for human tissue) by centrifugation. Immune cells were collected for further analysis.

### Flow cytometry and sorting

Single cell suspension was first stained with the fixable viability dye at 1:1000 in PBS for 10 min. After washing with flow activated cell sorting (FACS) buffer (PBS/2%FBS), cells were incubated with Fc block at 1:200 for 10 min, followed by staining with indicated antibody mixtures for 30 min before washing and flow cytometry analysis. For intracellular staining, including IgE, cells were fixed and permeabilized using the FoxP3 staining Buffer Set according to the manufacturer’s protocol. Cells were then incubated with Fc block and intracellular antibodies for 30 min before washing and flow cytometry analysis. All of the steps were performed at 4 °C. For intracellular cytokine analysis, cells were stimulated with the BD Leukocyte Activating Cocktail, with BD GolgiPlug for 5 h at 37 °C with 5% CO_2_, prior to staining, as described above. Cells were acquired on a BD LSR II or FACSymphony using FACSDiva software (BD Biosciences) and analyzed using FlowJo software (Treestar). For cell sorting, single cell suspensions isolated from spleens were enriched for CD4^+^ T-cells using the CD4 microbeads (Miltenyi Biotec). Enriched CD4^+^ T-cells or tumor cells were then stained with viability dye and surface antibodies as described above, followed by sorting on a FACSAria II using FACSDiva software.

### Adoptive transfer and tamoxifen treatment

Donor mice were i.p. immunized with 100 μg NP-OVA in CFA as described above. Splenocytes were isolated from donor mice and CD4^+^ T-cells were enriched using the CD4 microbeads before sorting CD4^+^PD-1^+^CXCR5^+^ follicular T-cells. 5 × 10^5^ sorted cells were intravenously injected into *Tcrα*^***−****/****−***^ mice followed by B16-OVA implantation and NP-OVA immunization, as described above. To deplete Blimp1 in T_FR_ cells, mice were i.p. injected with 1 mg tamoxifen emulsified in sunflower oil once every 24 h for 5 consecutive days. Mice were monitored daily after injection.

### Enzyme-linked Immunosorbent assay (ELISA)

In this study, peripheral blood (about 0.2 ml) was collected from each mouse at the experiment endpoint. Serum was separated via centrifugation and frozen at − 20 °C until testing. Total IgE titers were determined by the IgE OptEIA ELISA Set, according to the manufacturers’ protocol. Total IgG levels were measured using the purified goat anti-mouse IgG as the coating antibody, and goat anti-mouse IgG HRP as the detection antibody. The serum titers of anti-OVA IgE and anti-OVA IgG were measured using the OVA protein as the coating reagent, and biotinylated anti-mouse IgE followed by streptavidin HRP (contained in the IgE OptEIA ELISA Set) or goat anti-mouse IgG HRP as the detection antibody, respectively. The OD was read on the Ultra Micro EL 808 microplate reader (Biotek Instruments) at 450 nm.

### Immunofluorescent microscopy

Mouse tumor tissues were flash frozen in liquid nitrogen and embedded in optimum cutting temperature (OCT) compound. The frozen blocks were stored in − 80 °C until sectioning into 7 μm sections. Sections were stored in − 80 °C before being thawed and fixed with acetone for 10 min at − 20 °C. Sections were then blocked with either PBS/5% BSA or PBS/5% animal serum matching the species of the secondary fluorescent antibody for 1 h. Sections were stained with FITC-conjugated anti-mouse CD3ε and Alexa Fluor 594-conjugated rat anti-mouse CD45R/B220, or Alexa Fluor 647-conjugated rat anti-mouse CD31, or Alexa Fluor 488-conjugated rat anti-mouse CD68 and purified rat anti-mouse IgE that was visualized using Alexa Fluor 555-conjugated goat anti-rat IgG, or purified Armenian hamster anti-mouse FcεRIα that was visualized using Alexa Fluor 594-conjugated goat anti- hamster IgG. Nuclei were counterstained with DAPI. For analysis of T_FR_ and T_FH_ cells from formalin-fixed paraffin embedded (FFPE) melanoma tissues, tissue blocks were cut into 5–7 μm sections onto microscope slides followed by deparaffinizing and rehydrating. Sections then underwent antigen retrieval by boiling in IHC Antigen Retrieval Solution (high pH) before maintaining at a sub-boiling temperature for 20 min. After cooling and washing, sections were then blocked for any non-specific binding in PBS/Tween20/5% animal serum for 30 min at room temperature (RT) before staining with purified rabbit anti-human CD4, mouse anti-human CXCR5 and rat anti-human Foxp3 for 2 h at RT. After washing, Alexa Fluor 488-conjugated goat anti-rabbit IgG, Alexa Fluor 647-conjugated goat anti-mouse IgG and Alexa Fluor 555-conjugated goat anti-rat IgG were added respectively, and incubated for 1 h at RT prior to counterstaining nuclei with DAPI. Images were captured with a Leica DMRB microscope equipped with Hamatsu C4742-95 and 3CCD color cameras and appropriate filter cubes, and acquired using OpenLAB 3.1 software (Agilent Technologies). Image pseudo-coloring and quantification of stained areas or cells were performed using ImageJ software (NIH).

### In vitro Treg suppression assay

The suppression assay by Treg cells was performed as previously described [24]. Briefly, CD8^+^ T-cells were enriched from spleens using the CD8 microbeads, and Treg cells from spleens and tumors were enriched using the CD4^+^CD25^+^ Regulatory T-Cell Isolation Kit. Enriched CD8^+^ T-cells were then labelled with the cell trace violet (CTV) and cultured alone, or with enriched Treg cells from indicated tissue and mice plus 5 μg/ml plate-bound anti-CD3ε and 2 μg/ml anti-CD28. 60 h later, CTV staining of CD8^+^ T-cells was analyzed by flow cytometry. The division index was retrieved using the FlowJo proliferation tool and the percent of suppression was calculated as 100-(division index in each experimental group / division index in CD8^+^ T-cell alone group)*100.

### Isolation of mouse bone marrow-derived macrophages (BMDMs)

BMDMs were isolated and differentiated as previously described [25]. Bone marrow cells were flushed from the femurs and tibias of sacrificed mice using cold 1× PBS until the bones appeared clear. The cells were centrifuged and filtered through a 70 μm cell strainer and red blood cells lysed. Cells were cultured in complete DMEM containing 10 ng/mL M-CSF for 7 days with a media change every 2 days. Cultured cells were harvested and stained with the marker F4/80 to assess the purity before further analysis.

### Macrophage-mediated phagocytosis and killing of tumor cells

2 × 10^5^ CTV-labeled B16-OVA cells were co-cultured with 1 × 10^5^ differentiated BMDMs in complete DMEM along with 2.5 μl serum (1.25% of the total culture) collected from tumor-bearing mice for 2 h or overnight. Sera pre-treated with anti-IgE (50 μg/ml) were included as controls. Following co-culture, the frequency of CTV^+^F4/80^+^ macrophages and CTV^+^B16-OVA cells positive for the viability dye, defined as dead cells, were measured by flow cytometry, respectively. The percent tumor killing is calculated using the following equation: [%dead cells (experimental group)–%dead cells (tumor alone)]/% dead cells (tumor alone).

### TCGA dataset analysis

Correlation analyses of relevant genes with RSEM normalized TPM values from the TCGA-Skin Cutaneous Melanoma (SKCM) study were performed using Tier 3 standardized, normalized, batch corrected, and platform-corrected RNASEQ datasets downloaded from the Oncolnc server (http://www.oncolnc.org). Linear regression model was used to calculate correlation coefficients and *p* value. To analyze multi-gene dependent fractions between primary and metastatic groups and geneset scoring based survival proportions, normalized gene expression data and clinical information of each patient from the TCGA-SKCM dataset were downloaded using UCSC Xena (https://genome-cancer.ucsc.edu/). Gene signature-based score was calculated by averaging log transformed transcript levels of indicated genes as described elsewhere [26]. Survival time was chosen based on the overall survival (OS) time and OS status. The OS status denotes survival time in days, and the status indicates whether the patient’s death was observed (status = 1) or that survival time was censored (status = 0). To investigate whether the differences in geneset-based computed scores can influence a patient’s survival, patient samples were grouped into high scoring or low scoring cohorts based on their top or bottom percentile scores. Survival curves were computed for the patients with each group using the Kaplan-Meier method. The log rank test was used to define whether patients with different geneset scores have significantly different survival time (*p* < 0.05).

### RNA isolation and sequencing

Total RNA from sorted CD44^+^ Treg cells was extracted using a QIAshredder kit (QIAGEN) and an RNeasy Plus Micro Kit (QIAGEN) according to the manufacturer’s protocol. RNA sequencing was performed at Genewiz (South Plainfield, NJ) using Ultra-Low Input RNA-seq Service. Briefly, mRNA was specifically enriched from total RNA by removing rRNA and hydrolyzed into small pieces. The fragments were then reverse-transcribed into first-strand cDNA using random hexamer primers, followed by second strand synthesis. The short cDNA strands were ligated with 3′- and 5′-adapters for amplification and sequencing using the Illumina® platforms (HiSeq 2 × 150 bp, single index, per lane). The RNA-seq data have been deposited in the NCBI GEO under accession number GSE178135.

### RNA-seq data analysis

Sequence reads were trimmed to remove possible adapter sequences and nucleotides with poor quality using Trimmomatic v.0.36. The trimmed reads were mapped to the *Mus musculus* GRCm38 reference genome available on ENSEMBL using the STAR aligner v.2.5.2b. The STAR aligner is a splice aligner that detects splice junctions and incorporates them to help align the entire read sequences. BAM files were generated as a result of this step. Unique gene hit counts were calculated by using featureCounts from the Subread package v.1.5.2. Only unique reads that fell within exon regions were counted. After extraction of gene hit counts, the gene hit counts table was used for downstream differential expression analysis. Using DESeq2, a comparison of gene expression of each group was performed. The Wald test was used to generate *p*-values and log2 fold changes. Genes with an adjusted p-value < 0.05 and absolute log2 fold change > 1 were called as differentially expressed genes for each comparison. The Volcano plot shows the global transcriptional change across the groups compared. All the genes are plotted and each data point represents a gene. The log2 fold change of each gene is represented on the x-axis and the −log10 of its adjusted *p*-value is on the y-axis. The upregulated genes in WT eTreg cells with an adjusted p-value < 0.05 and a log2 fold change > 1 are indicated by red dots. The downregulated genes in WT eTreg cells with an adjusted p-value < 0.05 and a log2 fold change < − 1 are indicated by blue dots. Principal component analysis was performed to reveal the similarity within and between groups. Differentially expressed genes were also analyzed using Advaita’s “iPathawayGuide” (Advaita Bioinformatics) or g:Gost (g:Profiler) to reveal the biological pathways.

### NanoString nCounter mRNA analysis

Total RNA from sorted CD45^−^ tumor cells was extracted using a QIAshredder kit (QIAGEN) and an RNeasy Plus Micro Kit (QIAGEN) according to the manufacturer’s protocol. RNA quantification was performed using the DeNovix DS-11 Spectrophotometer (DeNovix, Inc). 100 ng of purified RNA was added to 3 μL of Reporter CodeSet and 2 μL Capture ProbeSet using an nCounter master kit as recommended (NanoString Technologies). Samples were processed on the NanoString nCounter Flex System per manufacturer instructions using the nCounter Mouse PanCancer Immune Profiling panel or the nCounter Mouse PanCancer Pathways panel (NanoString Technologies). Each gene set interrogates 750 cancer-related genes alongside 20 internal reference controls (full gene list and controls available on manufacturer’s website). Differentially expressed genes were identified in nSolver 4.0 Analysis Software (NanoString) as genes with a *p* value of less than 0.05 versus the respective baseline control. Reactome pathway analysis was performed using the NetworkAnalyst. The NanoString data have been deposited in the NCBI GEO under accession number GSE178135.

### Statistics

Statistical analyses were performed using two-tailed, unpaired or paired Student’s t-test, or two-way ANOVA with GraphPad Prism V8 software. Error bars indicate mean ± SEM. A *P* value of < 0.05 was considered to be statistically significant (*P < 0.05, **P < 0.01, ***P < 0.001, ****P < 0.0001). No exclusion of data points was used. Sample size was not specifically predetermined, but the number of mice used was consistent with previous experience with similar experiments.

## Results

### Blimp1^+^ Treg and T_FR_ cells are accumulated in the tumor

**Fig. 1:**
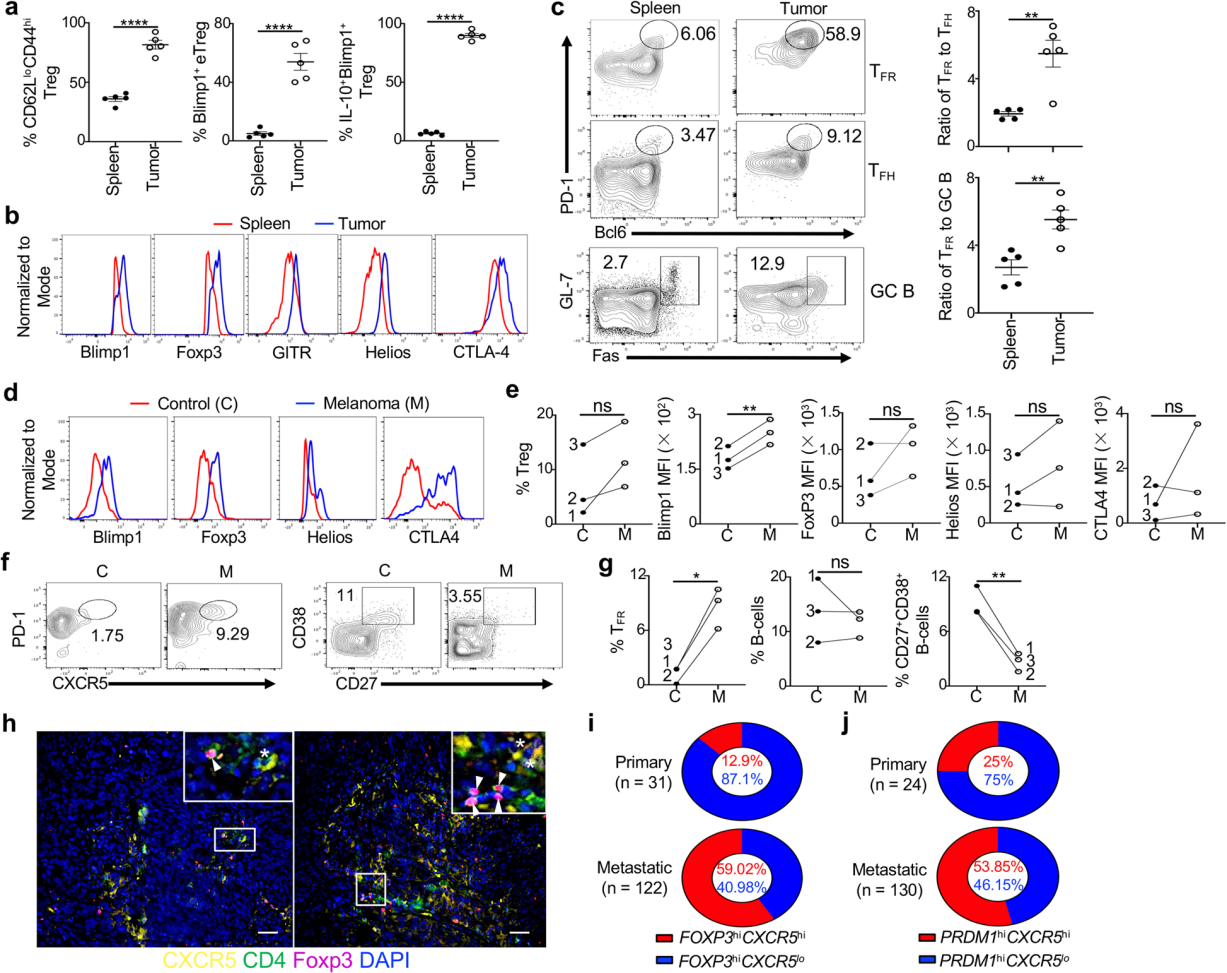
Blimp1^+^ Treg and T_FR_ cells are accumulated in the tumor. **a-b**) Blimp1-YFP reporter mice (*n* = 5) were inoculated with B16-OVA and immunized with NP-OVA/CFA at day 0 and NP-OVA/IFA at day 7. Cells from spleens and tumors were analyzed at day 21 by flow cytometry. Blimp1 = YFP. **a**) Frequencies of indicated subsets. eTreg: CD62L^lo^CD44^hi^Treg. **b**) Expression of indicated molecules in Blimp1^+^Foxp3^+^Treg cells. **c**) *Foxp3*^YFP-Cre^ mice (n = 5) were established with B16-OVA/NP-OVA model as in a. *left*, expression of T_FR_ (PD-1^+^Bcl6^+^Foxp3^+^CD4^+^CD3^+^), T_FH_ (PD-1^+^Bcl6^+^Foxp3^−^CD4^+^CD3^+^) and GC B-cells (GL-7^+^Fas^+^CD19^+^); *right*, Ratios of T_FR_:T_FH_ or T_FR_:GC B in the spleen or tumor. Data represent one of two (a-b) or at least three (c) independent experiments. **d**) Representative analysis of indicated molecules in Treg cells (CD25^+^CD127^−^CD4^+^CD3^+^) from melanoma metastatic lung (M) or uninvolved lung tissue (Control, C) of the same patient. **e**) Frequency of Treg cells and mean fluorescence intensity (MFI) of indicated molecules as presented in d. **f**) T_FR_ (PD-1^+^CXCR5^+^CD25^+^CD127^−^CD4^+^CD3^+^) and CD27^+^CD38^+^ B-cells (gated on IgD^−^CD19^+^CD3^−^) from melanoma metastatic lung (M) or uninvolved lung tissue (C) of the same patient. **g**) Frequency of each subset in f. Other melanoma metastatic tissues are included in e and g: 1, lung; 2, liver; 3, lymph node. **h**) IF staining of T_FR_ (CXCR5^+^Foxp3^+^CD4^+^) and T_FH_ (CXCR5^+^Foxp3^−^CD4^+^) cells in two FFPE melanoma metastatic lymph node sections (160 ×). Insets with a 4-fold magnification highlight T_FR_ (arrowheads) and T_FH_ (*) cells. **i-j**) Relative fractions, indicated by % with matched colors, of *CXCR5*^hi^ and *CXCR5*^lo^ (based on median expression value cutoff) patients within *FOXP3*^hi^ (**i**) or *PRDM1*^hi^ (**j**) group (top 33%) in primary and metastatic patients from the TCGA-SKCM dataset. ns, no significance, * *P* < 0.05, ** *P* < 0.01 and **** *P* < 0.0001 (a,c, unpaired two-tailed Student’s t-test; e,g, paired two-tailed Student’s t-test). Bars, mean ± SEM

To first understand the extent to which the TME could influence Blimp1^+^ Treg and T_FR_ cells, we analyzed Treg cells from Blimp1-YFP reporter mice inoculated with B16-OVA. To facilitate the analysis of T_FR_ cells and Ab response [10, 27], we immunized mice with NP-OVA, given that immunization or vaccination boosts the anti-tumor immune response [28]. When the tumor reached ~ 1 cm^3^, 80% TIL Treg cells but only 35% splenic Treg cells from an individual mouse displayed the eTreg phenotype (CD62L^lo^CD44^hi^Foxp3^+^CD4^+^) (Fig. 1a and Additional file 3a,b and 4a). Unlike splenic eTreg cells that only had 5% positive for Blimp1 (YFP), about 50% TIL eTreg cells expressed Blimp1 (YFP) and almost all of the TIL Blimp1^+^ Treg cells expressed IL-10, consistent with the role of Blimp1 in the regulation of IL-10 expression [7]. TIL Blimp1^+^(YFP^+^) Treg cells compared to their splenic counterparts also expressed higher levels of Blimp1, Foxp3, GITR, Helios and CTLA-4 (Fig. 1b and Additional file 4b), markers associated with an activated phenotype and robust suppressive activity [10, 29, 30]. Consistently, interrogating the transcriptome of TIL versus splenic CD44^+^ Treg cells (i.e., eTreg cells) that were isolated and sorted from these mice prior to RNA sequencing (RNA-seq) showed that TIL eTreg cells displayed a distinct transcriptional signature (Additional file 3c and 4c-d), suggesting that eTreg cells could adapt to the TME to acquire unique features.

Further analysis of these tumor-bearing mice revealed that T_FR_ cells (PD1^+^Bcl6^+^Foxp3^+^CD4^+^CD3^+^), T_FH_ cells (PD1^+^Bcl6^+^Foxp3^−^CD4^+^CD3^+^) and GC B-cells (GL7^+^Fas^+^CD19^+^) were more enriched in Foxp3^+^ Treg, CD4^+^Foxp3^−^ effector T-cells (Teff) and B-cell compartment, respectively, in the tumor than those in the spleen (Fig. 1c and Additional file 3a,b). A 2-3 fold increase in the ratios of T_FR_:T_FH_ or T_FR_:GC B in the tumor compared to those in the spleen suggested that the immunosuppression in the tumor might include suppression of T_FH_-Ab response by TIL T_FR_ cells.

### TIL Treg and T_FR_ cells from melanoma patients express higher levels of Blimp1 with increased suppressive phenotype

Next, we analyzed Treg and T_FR_ cells from a group of patients with stage IV melanoma (Additional file 2). The abundance of Treg cells (CD25^+^CD127^−^CD4^+^CD3^+^) in the metastatic tissues, including lung, liver and lymph node, were generally increased compared to Treg cells from adjacent uninvolved control tissues. These TIL Treg cells expressed higher levels of Foxp3, Helios and CTLA-4, except for slight decreases observed in the metastatic liver. Notably, these TIL Treg cells consistently expressed higher levels of Blimp1 than Treg cells from control tissues (Fig. 1d,e and Additional file 3d). Interestingly, there were substantially more T_FR_ cells (PD-1^+^CXCR5^+^CD25^+^CD127^−^CD4^+^CD3^+^) and fewer activated CD27^+^CD38^+^IgD^−^CD19^+^ B-cells in these metastatic tissues compared to control tissues, although the frequency of total B-cells was not significantly changed (Fig. 1f,g and Additional file 3d). The increased TIL T_FR_ cells and reduced activated B-cells appeared to be consistent throughout all of metastatic tissues, despite that not all tissues had the matched controls available (Additional file 5). Consistently, immunofluorescence (IF) analysis of FFPE metastatic lymph node sections revealed T_FR_ cells (CXCR5^+^Foxp3^+^CD4^+^) and T_FH_ cells (CXCR5^+^Foxp3^−^CD4^+^) with some of T_FH_ and T_FR_ cells being closely localized (Fig. 1h). This analysis suggested that TIL Treg and T_FR_ cells might be involved in the regulation of tumor immunity in a set of melanoma patients. Notably, analysis of a SKCM patient cohort from TCGA datasets showed that *PRDM1* mRNA expression was positively correlated with *FOXP3* mRNA expression, and among the *FOXP3*^hi^ population, *PRDM1* expression was positively correlated with the levels of *FUT4* and *FUT7*, genes encoding fucosyltransferase 4 and 7 that are enzymes responsible for the synthesis of CD15 and CD15s, respectively. The latter is a marker specific for highly suppressive human FOXP3^hi^ eTreg cells [31] (Additional file 4e-g). Moreover, there were increased proportions of metastatic patients with higher expression of *CXCR5* and *PRDM1* in Treg cells (Fig. 1i,j), suggesting that high levels of *CXCR5* and *PRDM1* in Treg cells are correlated with increased risk of melanoma metastasis.

### Delayed tumor growth and enhanced anti-tumor effector responses in mice with Foxp3-specific deletion of Blimp1

**Fig. 2:**
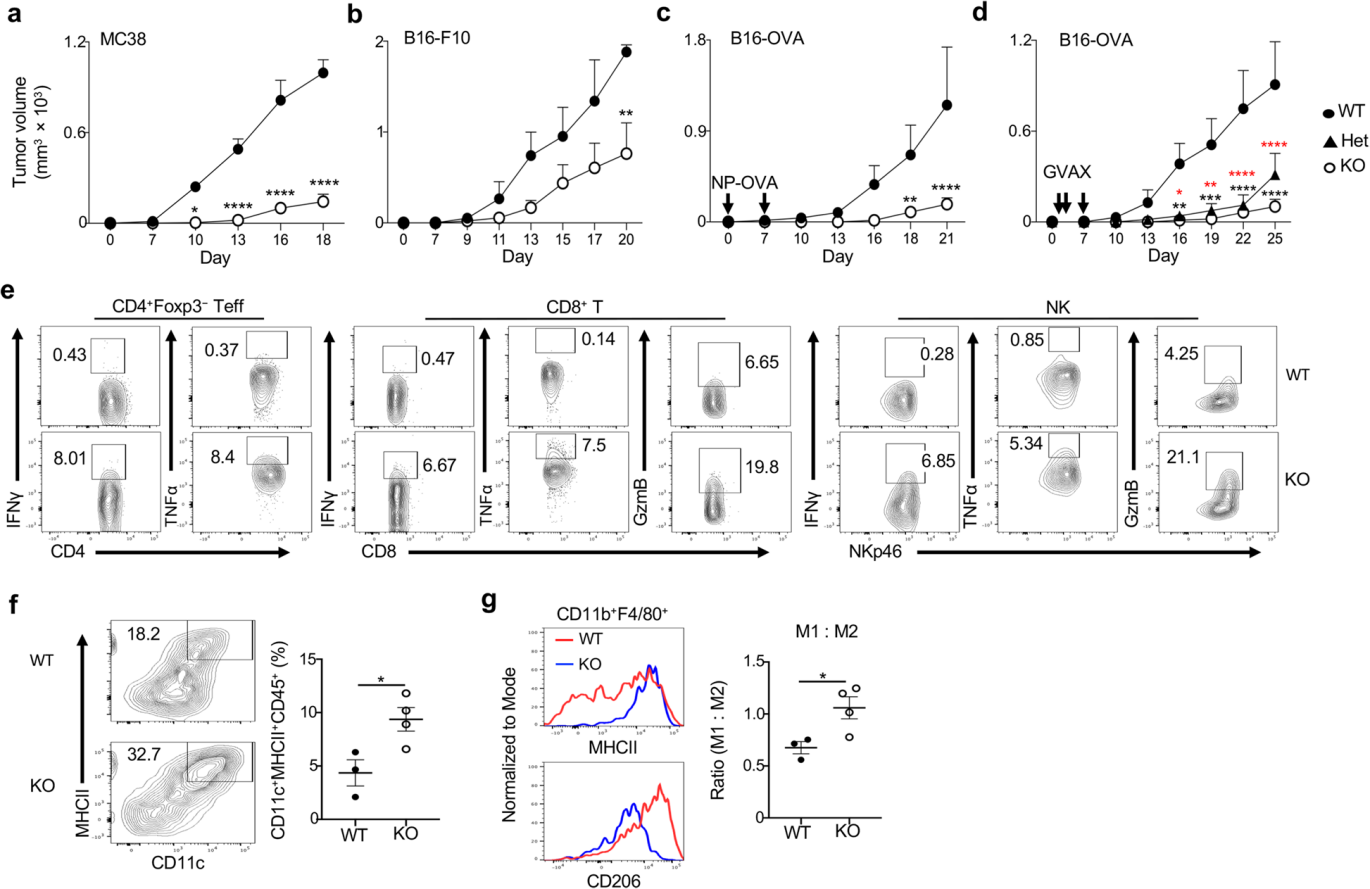
Delayed tumor growth and enhanced anti-tumor effector responses in mice with Foxp3-specific deletion of Blimp1. Blimp1 WT (*Foxp3*^YFP-Cre^), Het (*Prdm1*^fl/+^*Foxp3*^YFP-Cre^) and KO (*Prdm1*^fl/fl^*Foxp3*^YFP-Cre^) mice were inoculated with MC38 (*n* = 6 per group) (**a**), B16-F10 (WT: *n* = 4; KO: n = 6) (**b**) or B16-OVA at day 0 and immunized with NP-OVA at days 0, 7 (as Fig. 1a) (WT: n = 6; KO: *n* = 14) (**c**) or B16-OVA at day 0 and vaccinated with GVAX at days 1, 3 and 7 (WT: n = 4; Het: n = 4; KO: n = 6) (**d**), and tumor growth was monitored. **e**) Analysis of IFNγ, TNFα and GzmB expression in TIL CD4^+^Foxp3^−^ Teff, CD8^+^ T-cells and NK cells from mice as in c. **f**) Analysis and quantitation of TIL CD11c^+^MHCII^+^ DC (WT: *n* = 3; KO: n = 4). **g**) Expression of MHCII and CD206 on TIL F4/80^+^ macrophages, and ratio of MHCII^+^M1:CD206^+^M2 (WT: n = 3; KO: n = 4). Data represent one of two (a-b,d,f-g) or at least three (e) or are pooled from two (c) independent experiments. * *P* < 0.05, ** *P* < 0.01, *** *P* < 0.001 and **** *P* < 0.0001 (a-d, two-way ANOVA with sidak’s comparisons test (a-c) or with tukey’s test (d), black: KO compared to WT, red: Het compared to WT; f,g, unpaired two-tailed Student’s t-test). Bars, mean ± SEM

The above analysis of melanoma patients and mouse B16 models prompted us to investigate if Blimp1 expression in Treg and T_FR_ cells regulates tumor immunity. We then implanted B16 or MC38 cells into *Foxp3*^YFP-Cre^ (WT) mice and mice harboring a deletion of *Prdm1* in Foxp3^+^ T-cells, which mainly affects eTreg subsets (*Prdm1*^fl/fl^*Foxp3*^YFP-Cre^) [10]. While B16.F10 and MC38 tumors gradually developed in WT mice, *Prdm1*^fl/fl^*Foxp3*^YFP-Cre^ mice had delayed tumor growth with smaller volumes (Fig. 2a,b). As expected, immunization with NP-OVA or vaccination with irradiated GVAX [20] delayed tumor growth in both groups. However, *Prdm1*^fl/fl^*Foxp3*^YFP-Cre^ mice remained to develop much smaller tumors with a slower growth rate than WT mice, while a partial deletion of Blimp1 in heterozygous *Prdm1*^fl/+^*Foxp3*^YFP-Cre^ mice also prevented the tumor growth (Fig. 2c,d). Immune profiling revealed that tumor-infiltrating, but not splenic, CD4^+^Foxp3^*−*^Teff, CD8^+^ T-cells and NK cells expressed markedly higher levels of effector molecules, including IFNɣ, TNFα and Granzyme B (GzmB), in *Prdm1*^fl/fl^*Foxp3*^YFP-Cre^ mice compared to WT mice, despite that the total frequency of these cells in the tumor was not significantly changed (Fig. 2e and Additional file 6a-c). In addition to these effector cells, dendritic cells (DC) (CD11c^+^MHCII^+^) and MHCII^+^ M1 type macrophages relative to CD206^+^ M2 type macrophages (CD11b^+^GR-1^−^F4/80^+^) (but not the frequency of total macrophages) in the tumor of *Prdm1*^fl/fl^*Foxp3*^YFP-Cre^ mice were increased compared to WT mice (Fig. 2f,g and Additional file 6a). Taken together, these results suggested that the deletion of Blimp1 in Treg cells resulted in improved tumor control associated with enhanced activation of both adaptive and innate effector cells in the tumor.

### TIL Blimp1-deficient Treg cells convert into effector T-cells and display impaired suppressive activity

**Fig. 3:**
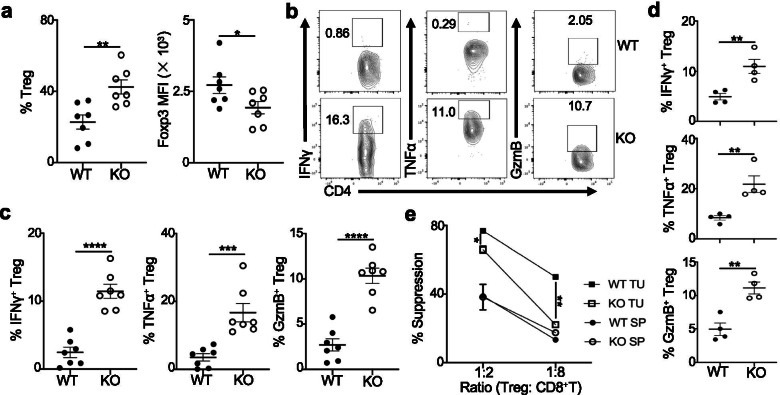
TIL Blimp1-deficient Treg cells convert into effector T-cells and display impaired suppressive function. **a-c**) Analysis of TIL Treg cells (*n* = 7 per group) from mice as in Fig. 2c. Quantitation of frequency and Foxp3 MFI of Treg cells (**a**); IFNγ, TNFα and GzmB expression in Treg cells (**b**) or frequency of TIL Treg cells expressing these molecules (**c**). **d**) B16-OVA/GVAX model was established as in Fig. 2d. IFNγ, TNFα and GzmB expression in TIL Treg cells (n = 4 per group). **e**) In vitro suppression of CTV-labelled CD8^+^ T-cell proliferation performed in duplicates per experimental group. Percent suppression is shown. WT: *Foxp3*^YFP-Cre^, KO: *Prdm1*^fl/fl^*Foxp3*^YFP-Cre^. SP: spleen; TU: tumor. Data represent one of at least three (a-c) or two (d-e) independent experiments. * *P* < 0.05, ** *P* < 0.01, *** *P* < 0.001 and **** *P* < 0.0001 (a,c,d,e, unpaired two-tailed Student’s t-test). Bars, mean ± SEM

We next analyzed the Treg compartment from these tumor-bearing mice. Despite an increased frequency, TIL Treg cells from *Prdm1*^fl/fl^*Foxp3*^YFP-Cre^ mice downregulated Foxp3 but expressed increased effector molecules (IFNγ, TNFα and GzmB) compared to WT Treg cells (Fig. 3a-c), suggesting that TIL Blimp1-deficient Treg cells were unstable and reprogrammed into Teff. The conversion of TIL Treg cells was also observed in *Prdm1*^fl/fl^*Foxp3*^YFP-Cre^ mice vaccinated with GVAX (Fig. 3d). In contrast, there were no significant differences in the frequencies and effector molecule expression comparing splenic Treg cells from *Prdm1*^fl/fl^*Foxp3*^YFP-Cre^ mice to WT mice (Additional file 6d), indicating that conversion of Blimp1-deficient Treg cells into Teff was largely restricted to the tumoral compartment.

The conversion of TIL Blimp1-deficient Treg cells may reflect a loss of suppressive activity by these cells. To test this proposition, we performed the in vitro suppression assays using Treg cells isolated from tumors compared to spleens from both groups of mice. Coculture of splenic Treg cells with CTV-labelled CD8^+^ T-cells showed that these Treg cells from both mice equally suppressed CD8^+^ T-cell proliferation. TIL Treg cells exhibited more suppressive activity than splenic Treg cells, and TIL Treg cells from WT mice were able to suppress CD8^+^ T-cell proliferation. However, TIL Treg cells from *Prdm1*^fl/fl^*Foxp3*^YFP-Cre^ mice had greatly reduced suppression on CD8^+^ T-cell proliferation (Fig. 3e). These findings suggested that only Treg cells in the tumor were converted and no longer efficiently suppressed Teff.

### Foxp3 specific deletion of Blimp1 results in increased anti-tumor humoral immunity

**Fig. 4:**
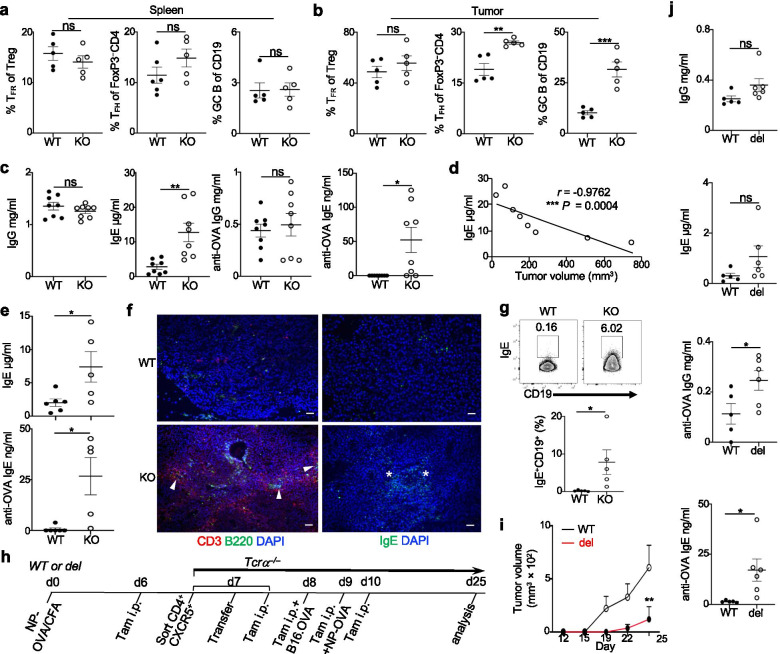
Foxp3 specific deletion of Blimp1 results in increased anti-tumor humoral immunity. Analysis was performed on splenic or tumoral cells from mice bearing B16-OVA (as in Fig. 2c). Frequency of T_FR_, T_FH_ and GC B-cells (as gated in Fig. 1c) in spleen (**a**) or tumor (**b**) (n = 5 per group). **c**) Serum total IgG, IgE and anti-OVA IgG, IgE titers (*n* = 8 per group). **d**) The relationship of serum IgE titers and tumor sizes in KO mice. **e**) B16-OVA/GVAX model was established as in Fig. 2d. Serum total IgE and anti-OVA IgE titers (WT: n = 6; KO: n = 5). The value 0 is used to indicate the undetectable anti-OVA IgE titers in c,e. **f**) Representative IF staining of T (CD3), B (B220), or IgE in the tumor (100 ×). Arrowheads, T/B clusters; *, IgE. **g**) Intracellular staining and quantitation of IgE in CD19^+^ B-cells (n = 5 per group). WT: *Foxp3*^YFP-Cre^, KO: *Prdm1*^fl/fl^*Foxp3*^YFP-Cre^. **h-j**) Transfer of Blimp1-deficient T_FR_ cells induces better anti-tumor response. WT: *Rosa26*^ERT2-Cre^; del: *Prdm1*^fl/fl^*Rosa26*^ERT2-Cre^. **h**) Schematic diagram of experiment. **i**) Tamoxifen-induced deletion of Blimp1 in T_FR_ cells delayed tumor growth (n = 3 per group). **j**) Serum antibody titers (WT: n = 5; del: n = 6). Data represent one of at least three (a-d, f) or two (e,g,i) or are pooled from two (j) independent experiments. ns, no significance, * *P* < 0.05, ** *P* < 0.01 and *** *P* < 0.001 (a,b,c,e,g,j, unpaired two-tailed Student’s t-test; d, Spearman’s *r*; i, two-way ANOVA with Sidak’s comparisons test). Bars, mean ± SEM

We next analyzed T_FR_, T_FH_ and GC B-cells in *Prdm1*^fl/fl^*Foxp3*^YFP-Cre^ and WT mice bearing B16-OVA tumors. Compared to WT mice, *Prdm1*^fl/fl^*Foxp3*^YFP-Cre^ mice had increased frequency of T_FH_ and GC B-cells in the tumor; this difference was not observed in the spleen and there was no significant difference of T_FR_ cell frequency in the spleen and tumor from both groups of mice (Fig. 4a,b). Although there were high titers of serum IgG and anti-OVA (tumor) IgG Abs, only slight increases were observed in *Prdm1*^fl/fl^*Foxp3*^YFP-Cre^ mice. In contrast, *Prdm1*^fl/fl^*Foxp3*^YFP-Cre^ mice had significantly elevated titers of serum IgE and anti-OVA (tumor) IgE Abs compared to WT mice, and anti-OVA IgE Abs were almost undetectable in WT mice (Fig. 4c). Consistent with the finding that serum specific IgE scores are inversely correlated with the risk of melanoma [32], serum IgE titers were inversely correlated with the tumor sizes of *Prdm1*^fl/fl^*Foxp3*^YFP-Cre^ mice (Fig. 4d). The increased IgE and anti-OVA (tumor) IgE Ab titers were also observed in *Prdm1*^fl/fl^*Foxp3*^YFP-Cre^ mice vaccinated with GVAX (Fig. 4e). IF analysis further confirmed that more CD3^+^ and B220^+^ cells were accumulated in the tumor of *Prdm1*^fl/fl^*Foxp3*^YFP-Cre^ mice. Compared to the diffused distribution of these cells in WT mice, more of these cells were clustered in the tumor of *Prdm1*^fl/fl^*Foxp3*^YFP-Cre^ mice (Fig. 4f and Additional file 7a). Interestingly, we also observed increased IgE deposition in the tumor along with increased IgE^+^ B-cells in these mice compared to WT mice (Fig. 4f,g and Additional file 7b). Taken together, these results demonstrated how loss of suppressive function by Blimp1^+^ Treg cells could lead to an increased cellular and humoral anti-tumor response, thus resulting in better tumor control. Despite the robust anti-tumor responses, no obvious autoimmune phenotype was observed throughout the experiments.

### Transfer of Blimp1-deficient T_FR_ cells induces better anti-tumor response

Blimp1^+^ Treg cells comprise both T_FR_ cells and conventional non-T_FR_ Treg cells. Our recent publication has indicated that Blimp1-deficient non-T_FR_ Treg cells do not contribute significantly to the increased frequency of T_FH_ and GC B-cells or dysregulated Ab responses observed in *Prdm1*^fl/fl^*Foxp3*^YFP-Cre^ mice [10]. Instead, Blimp1-deficient T_FR_ cells are capable of supporting GC-Ab response due to the acquisition of T_FH_-like properties post-immunization [10]. To further define the contribution of Blimp1^+^ T_FR_ cells independent of other Treg cells to the regulation of Ab responses and tumor growth, we used an inducible Blimp1 deletion system to circumvent potential developmental defects secondary to inflammation or other changes in the environment (Fig. 4h). We generated *Prdm1*^fl/fl^*Rosa26*^Cre-ERT2^ (del) mice to allow deletion of Blimp1 after administration of tamoxifen. Follicular T-cells (PD1^+^CXCR5^+^CD4^+^) (both Blimp1^+^ T_FR_ and Blimp1^−^ T_FH_) were sorted from *Prdm1*^fl/fl^*Rosa26*^Cre-ERT2^ mice or *Rosa26*^Cre-ERT2^ (WT) mice 7 days after NP-OVA immunization and 1 day after tamoxifen administration. These cells were then transferred into *Tcra*^−/−^ mice before B16-OVA implantation and injection of tamoxifen for 4 additional days (Fig. 4h,i). This method can substantially reduce Blimp1 expression specifically by T_FR_ cells from *Prdm1*^fl/fl^*Rosa26*^Cre-ERT2^ mice along with increased T_FR_, T_FH_ and GC B-cells [10]. We observed that *Tcrα*^−/−^ mice transferred with follicular T-cells bearing Blimp1-deleted T_FR_ cells had smaller and delayed tumor growth associated with increased total and particularly OVA-specific IgG and IgE compared to mice transferred with follicular T-cells containing WT T_FR_ cells (Fig. 4j). The results obtained from this adoptive transfer assay suggested that transfer Blimp1-deleted T_FR_ cells compared to WT T_FR_ cells can intrinsically contribute to increased Ab production and enhanced tumor control.

### Sera from *Prdm1*^fl/fl^*Foxp3*^YFP-Cre^ tumor-bearing mice increase the anti-tumor activity of macrophages

**Fig. 5:**
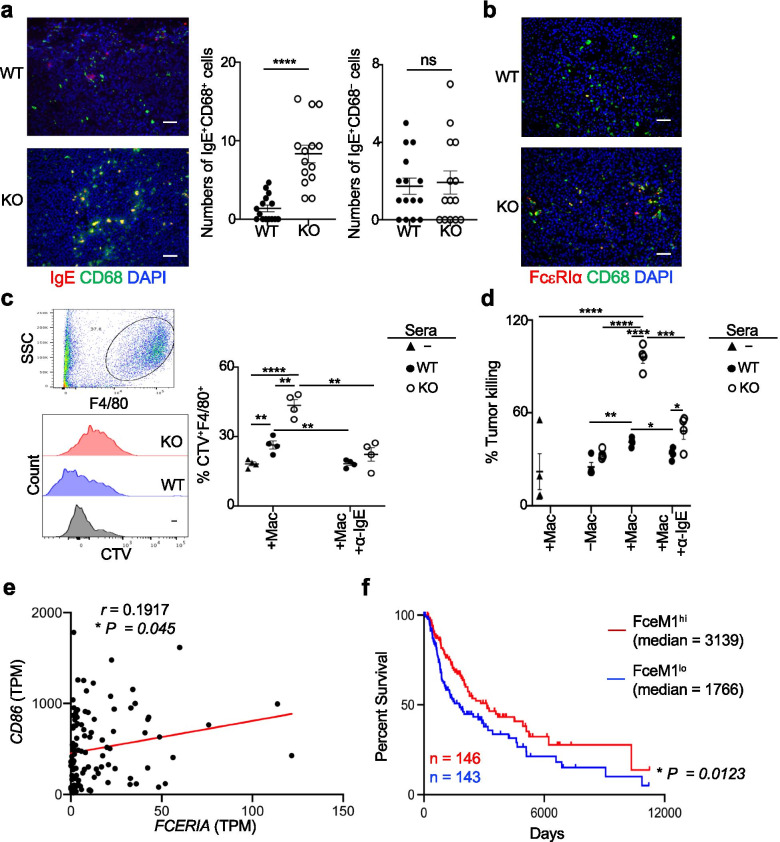
Sera from *Prdm1*^fl/fl^*Foxp3*^YFP-Cre^ tumor-bearing mice increase the anti-tumor activity of macrophages. **a-b**) B16-OVA model was established as in Fig. 2c. IF staining of IgE and CD68 in the tumor and quantitation of IgE^+^CD68^+^ and IgE^+^CD68^−^ cells (**a**); or IF staining of FcεR1α and CD68 in the tumor (**b**). Each dot represents the counted numbers within a field of view (160 ×). WT: *n* = 15 views from 6 mice; KO: n = 14 views from 6 mice. **c-d**) BMDMs were co-cultured with CTV-labelled B16-OVA cells treated with or without a same volume of sera (2.5 μl, 1.25% of the total culture) collected from WT or *Prdm1*^fl/fl^*Foxp3*^YFP*-*Cre^ (KO) tumor-bearing mice for 2 h (**c**) or overnight (**d**) in quadruplicates. In a group, sera were pre-treated with anti-IgE (α-IgE). **c**) BMDMs with phagocytosed B16-OVA cells were indicated as CTV^+^F4/80^+^ cells (*upper left*, representative F4/80^+^ plot; *lower left*, representative histogram of CTV expression gated on F4/80^+^ cells; *right*, quantitation). **d**) Percent tumor killing is shown after quantifying dead tumor cells (CTV^+^ cells positive for viability-dye). Tumor cells alone treated with sera were included as controls. Triangles, tumor cells and BMDMs with no sera added. ns, no significance, * *P* < 0.05, ** *P* < 0.01, *** *P* < 0.001 and **** *P* < 0.0001 (a,c,d, unpaired two-tailed Student’s t-test). Bars, mean ± SEM. **e**) The correlation of *CD86* and *FCER1A* expression in top 25% *CD68*^*hi*^ SKCM patients from the TCGA dataset (*n* = 110) was analyzed by Pearson correlation (two-tailed, no adjustment for multiple comparisons because of one correlation test for a gene pair). The values of the coefficients (r) and significance (p) are indicated. **f**) Kaplan-Meier analysis of OS of patient cohorts expressing differential FceM1 signature (top 33% vs bottom 33%) based on combined log-averaging of *CD68*, *CD86* and *FCER1A* transcript levels from the TCGA-SKCM dataset. *P* value is generated using two-tailed LogRank test. Median, median survival time

Antibodies specific to tumor antigens bind directly to tumor cells while cross-linking with Fc receptors on effector cells, which triggers antibody dependent cell-mediated cytotoxicity or phagocytosis of tumor cells. The Ab deposition in the tumor may contribute to effector cell-mediated tumor killing, and IgE has been reported to promote the macrophage polarization into M1 phenotype [33] and modulate macrophages against cancer [34, 35], while we noted increased intratumoral M1 macrophages in *Prdm1*^fl/fl^*Foxp3*^YFP-Cre^ mice (Fig. 2g). To identify how increased IgE in the tumor could impact the cellular immunity against tumor cells, we first performed IF analysis of the IgE localization in relationship to macrophages in the tumor. Co-staining of the macrophage marker CD68 with IgE or its receptor FcεRIα revealed increased IgE^+^CD68^+^ and FcεRIα^+^CD68^+^ cells in the tumor of *Prdm1*^fl/fl^*Foxp3*^YFP-Cre^ mice compared to WT mice (Fig. 5a,b). Moreover, among all IgE^+^ cells in *Prdm1*^fl/fl^*Foxp3*^YFP-Cre^ tumors, over 85% cells were positive for CD68 while fewer CD68^−^ cells were co-stained with IgE, suggesting that macrophages were the major IgE effector cells. We then explored if increased Ab production in these mice may promote macrophage-mediated phagocytosis and/or killing of tumor cells using an in vitro culture assay. BMDMs from adult WT mice were co-cultured with CTV-labelled B16-OVA cells treated with or without a same volume of sera collected from *Prdm1*^fl/fl^*Foxp3*^YFP*-*Cre^ or WT tumor-bearing mice. Using a published flow cytometry-based measurement of in vitro phagocytosis by macrophages, where macrophages containing CTV-labelled tumor cells were analyzed [36], we noted that the addition of sera increased F4/80^+^CTV^+^ cells and there were more of these cells after treatment with *Prdm1*^fl/fl^*Foxp3*^YFP*-*Cre^ sera than WT sera (Fig. 5c), suggestive of increased phagocytosis by macrophages. Correspondingly, the highest killing was observed for tumors incubated with both *Prdm1*^fl/fl^*Foxp3*^YFP*-*Cre^ sera and macrophages (Fig. 5d). Importantly, neutralizing IgE activity by pre-incubating sera with anti-IgE markedly diminished macrophage-mediated phagocytosis and tumor killing (Fig. 5c,d). These findings suggested that sera from *Prdm1*^fl/fl^*Foxp3*^YFP*-*Cre^ mice, at least partly due to 4.5 fold more IgE included (Fig. 4c), had the potential to increase macrophage-mediated phagocytosis and killing of tumor cells, which may contribute to delayed tumor growth. Further analysis of TCGA datasets revealed that *FCERIA* was positively correlated with the M1 macrophage marker *CD86* within the *CD68*^hi^ population (Fig. 5e). A quantitative measure of the putative anti-tumor macrophage signature based on transcript levels of 3 factors extracted from the TCGA dataset, *CD68*, *CD86* and *FCERIA*, further showed that the higher expression of this signature (named as FceM1) was correlated with the better survival of SKCM patients (Fig. 5f).

### TIL eTreg cells from *Prdm1*^fl/fl^*Foxp3*^YFP-Cre^ mice display distinct transcriptomic profiles

**Fig. 6:**
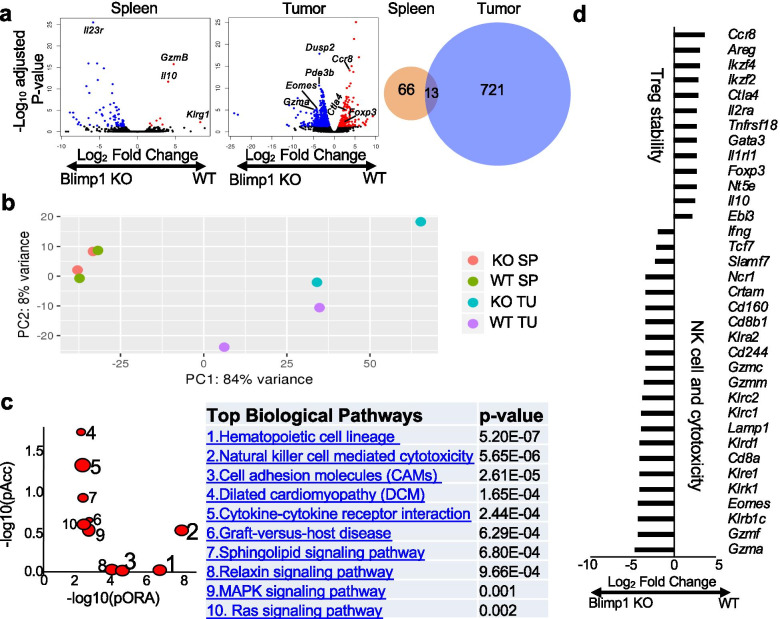
TIL eTreg cells from *Prdm1*^fl/fl^*Foxp3*^YFP-Cre^ mice display distinct transcriptomic profiles. WT or *Prdm1*^fl/fl^*Foxp3*^YFP-Cre^ (KO) mice were implanted with B16-OVA and immunized with NP-OVA (as in Fig. 2c). Splenic and tumoral eTreg cells (CD45^+^CD44^+^YFP^+^CD4^+^CD3^+^) were sorted for gene expression profiling (duplicates). **a**) DEGs in splenic and TIL WT and Blimp1 KO eTreg cells were analyzed by volcano plots and venn diagram. In the plots, each data point represents a gene. The x-axis and y-axis represent the log2 fold change of each gene and the -log10 of its adjusted *p*-value, respectively. Red dot: the upregulated genes in WT eTreg cells with an adjusted p-value < 0.05 and a log2 fold change > 1. Blue dots: the downregulated genes in WT eTreg cells with an adjusted p-value < 0.05 and a log2 fold change < − 1. Some genes involved in the regulation of Treg cells are denoted. **b**) Principal component analysis of all 4 subsets. SP: spleen; TU: tumor. **c**) Pathway enrichment of TIL WT and KO eTreg cells is presented by scatter plot and top biological pathways. pORA: probability of over-representation; pAcc: probability of accumulation. *Right*, *P* values are listed in the accompanying table. **d**) List of DEGs (at a > 2 log_2_-fold change and *P* < 0.05) of TIL WT and KO eTreg cells related to Treg stability or NK cell and cytotoxicity

To understand why the Treg alteration in *Prdm1*^fl/fl^*Foxp3*^YFP-Cre^ mice was selectively induced in the tumor but not in the periphery, we performed RNA-seq analysis of splenic and TIL CD44^+^ Treg cells from WT compared to *Prdm1*^fl/fl^*Foxp3*^YFP-Cre^ mice in our B16-OVA/NP-OVA model (Fig. 6a and Additional file 3c). While only 78 genes (at a > 2 log_2_-fold change and *P* < 0.05) were differentially expressed in splenic Blimp1-deficient compared to WT eTreg cells, 734 genes were differentially expressed in TIL Blimp1-deficient compared to WT eTreg cells and only 13 of these differentially expressed genes (DEGs) were shared in splenic and TIL eTreg cells (Fig. 6a), suggesting that the extent of differential gene expression imposed by Blimp1 deficiency in eTreg cells depended greatly on the tissue microenvironment. Principal component analysis of the relationship of these splenic and tumoral eTreg cells also revealed that TIL eTreg cells formed groups that were mostly distant from splenic eTreg cells irrespective of Blimp1 genotype (Fig. 6b). Moreover, the segregation of TIL Blimp1-deficient from WT eTreg cells was much greater than that of splenic eTreg cells, despite that variations existed for the TIL eTreg samples (Fig. 6b). Interestingly, pathway analysis further revealed that genes related to the NK mediated cytotoxicity pathway were highly represented in TIL Blimp1-deficient eTreg cells (Fig. 6c). Zooming in genes that were significantly differentially expressed in TIL Blimp1-deficient compared to WT eTreg cells showed that TIL Blimp1-deficient eTreg cells not only downregulated genes reflecting Treg stability and suppressive activity (e.g., *Foxp3*, *Ctla4*, *Il2ra*, *Ikzf2, Il10, Ebi3, Ikzf4*) but also concomitantly upregulated genes characteristic of NK cell cytotoxic program (e.g., *Eomes*, *Klrk1*, *Lamp1*, *Klrc2*, *Crtam*, *Ifng* and genes encoding granzymes) (Fig. 6d). Consistent with the reduced expression of *Il2ra* and Foxp3 (Fig. 3a), there were reduced levels of phospho-Stat5 (pStat5), but not phospho-Smad2/3 (pSmad2/3), in TIL Blimp1-deficient Treg cells compared to WT Treg cells (Additional file 8). The tissue-specific genetic profile suggested that TIL eTreg stable phenotype could be influenced by both Blimp1 expression and the TME.

### Deletion of Eomes in Blimp1-deficient Treg cells promotes tumor growth

**Fig. 7:**
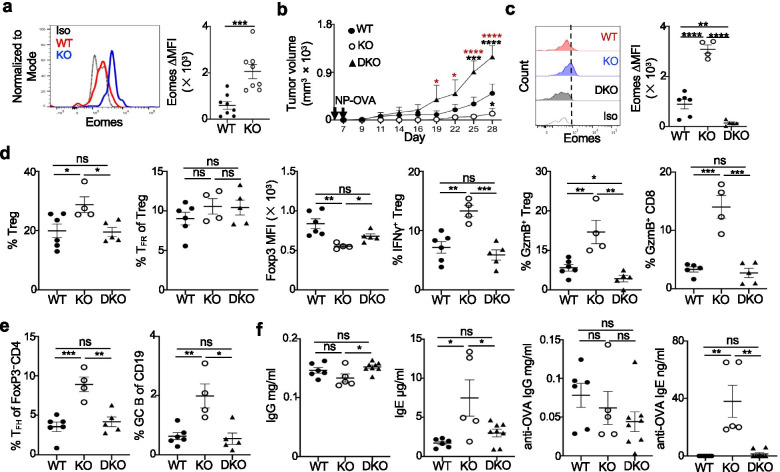
Deletion of Eomes in Blimp1-deficient Treg cells promotes tumor growth. **a**) Comparison and quantitation of Eomes levels in TIL Treg cells from B16-OVA/NP-OVA mice (n = 8 per group), as in Fig. 2c. **b**) B16-OVA model was established in each mouse strain (WT: n = 7; KO: n = 6; DKO: n = 5), as in Fig. 2c. Tumor sizes are shown. **c**) Comparison ad quantitation of Eomes MFI in TIL Treg cells from b. The vertical dotted line represents the threshold for the gating of Eomes^+^ cells. **d**) Frequency of TIL Treg and T_FR_ cells (PD-1^+^Bcl6^+^Foxp3^+^CD4^+^CD3^+^) and Foxp3 MFI of Treg cells as well as TIL Treg cells expressing IFNγ and GzmB or CD8^+^ T-cells expressing GzmB. **e**) Frequency of TIL T_FH_ (PD-1^+^Bcl6^+^Foxp3^−^CD4^+^CD3^+^) and GC B-cells (GL-7^+^Fas^+^CD19^+^). **f**) Serum total IgG, IgE and anti-OVA IgG, IgE titers. The value 0 is used to indicate the undetectable anti-OVA IgE titers. In c-f, WT: n = 5-6; KO: n = 4-5; DKO: n = 5-8. Iso: Isotype control. WT: *Foxp3*^YFP-Cre^, KO: *Prdm1*^fl/fl^*Foxp3*^YFP-Cre^, DKO: *Eomes*^fl/fl^*Prdm1*^fl/fl^*Foxp3*^YFP-Cre^. ∆MFI: MFI subtracted from the MFI of isotype controls. Data are pooled from two (a) or represent one of two independent experiments (b-f). ns, no significance, * *P* < 0.05, ** *P* < 0.01, *** *P* < 0.001 and **** *P* < 0.001 (a,c,d-f, unpaired two-tailed Student’s t-test; b, two-way ANOVA with Tukey’s comparisons test, black: compared to WT, red: compared to KO). Bars, mean ± SEM

We noted that the TF Eomes was highly upregulated in TIL Blimp1-deficient eTreg cells compared to WT eTreg cells (Figs. 6d and 7a). While Foxp3^+^ Treg cells express very low levels of Eomes and deletion of Eomes in Treg cells does not appear to affect their suppressive phenotype [37], Eomes is known to modulate the cytotoxic programs in Teff, including CD4^+^ cytotoxic T-cells [38]. Blimp1 can directly bind to the *Eomes* loci and regulate its expression [39]. We reasoned that the increased anti-tumor activity and cytotoxicity-like genetic program in TIL Blimp1-deficient eTreg cells may be at least in part mediated by the upregulation of Eomes. To test this proposition, we generated *Eomes*^fl/fl^*Prdm1*^fl/fl^*Foxp3*^YFP-Cre^ DKO mice with the dual deletion of Eomes and Blimp1 in Treg cells, and then established the B16-OVA/NP-OVA model in these mice and *Prdm1*^fl/fl^*Foxp3*^YFP-Cre^ as well as *Foxp3*^YFP-Cre^ (WT) mice (Fig. 7b,c). The upregulation of Eomes in TIL Blimp1-deficient Treg cells was almost abolished in TIL Treg cells of DKO mice, and consistently, *Prdm1*^fl/fl^*Foxp3*^YFP-Cre^ mice had delayed and smaller tumor growth than WT mice (Fig. 7b,c). Notably, deletion of Eomes in Blimp1-deficient Treg cells expedited and enhanced tumor growth, even in a greater extent than WT mice. Analysis of TIL Treg cells revealed that DKO mice had Treg cells at a similar frequency as WT mice and expressed increased levels of Foxp3 compared to Treg cells from *Prdm1*^fl/fl^*Foxp3*^YFP-Cre^ mice, albeit at a lower level than WT Treg cells (Fig. 7d). Correspondingly, the increased expression of GzmB in TIL CD8^+^ T-cells and the increased expression of IFNγ and GzmB in TIL Treg cells of *Prdm1*^fl/fl^*Foxp3*^YFP-Cre^ mice were greatly reduced in TIL of DKO mice, and GzmB expression in DKO TIL Treg cells was even lower than WT TIL Treg cells (Fig. 7d). Although the T_FR_ frequency did not change substantially, there were significantly reduced T_FH_ and GC B-cells in DKO TIL compared to TIL of *Prdm1*^fl/fl^*Foxp3*^YFP-Cre^ mice (Fig. 7d,e). Accordingly, the serum titers of total IgE and anti-OVA IgE in DKO mice were decreased to levels as WT mice, despite that an increase of total IgG and unaltered anti-OVA IgG levels comparing DKO to *Prdm1*^fl/fl^*Foxp3*^YFP-Cre^ mice were also observed (Fig. 7f). Taken together, these results suggested that deletion of Eomes in Blimp1-deficient Treg cells prevented their reprogramming and restored their suppressive phenotype, which may at least partly contribute to the tampered anti-tumor response and greater tumor growth.

### Deletion of Blimp1 in Treg cells remodels the TME and sensitizes the tumors to anti-PD-1 treatment

**Fig. 8:**
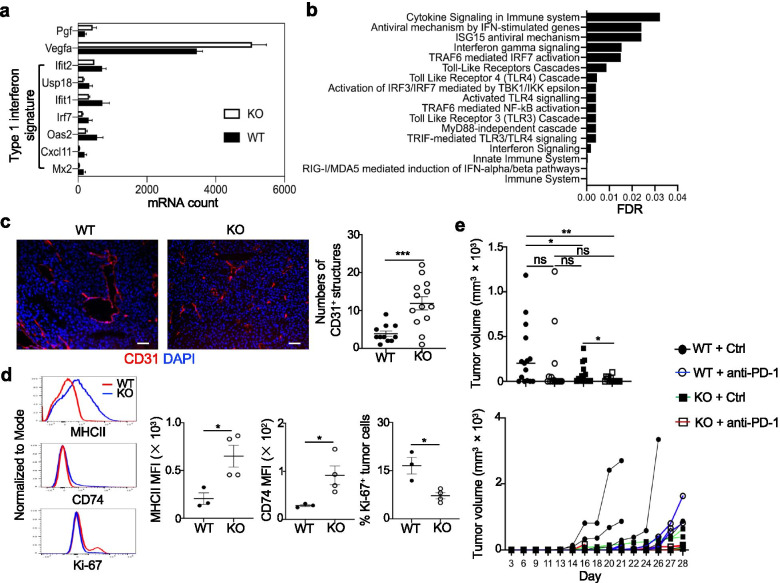
Deletion of Blimp1 in Treg cells remodels the TME and sensitizes the tumors to anti-PD-1 treatment. **a-d**) B16-OVA model was established as in Fig. 2c. **a-b**) CD45^−^ cells were sorted and subject to NanoString analysis. **a**) DEGs related to angiogenesis and type 1 IFN signature (≥ 1.5 fold change and *P* < 0.05) in *Foxp3*^YFP-Cre^ (WT) (n = 3) and *Prdm1*^fl/fl^*Foxp3*^YFP-Cre^ (KO) mice (*n* = 2). **b**) Reactome pathway analysis of DEGs revealed in a via NetworkAnalyst. **c**) IF staining of CD31 in the tumor and quantitation of CD31^+^ structures. Each dot represents the counted numbers within a field of view (160 ×). WT: n = 11 views from 6 mice; KO: *n* = 13 views from 6 mice. **d**) Expression and quantitation of MFI for MHCII and CD74 and percent Ki-67 of CD45^−^ cells (WT: n = 3; KO: n = 4). **e**) Mice (WT + Ctrl, WT + a-PD-1, KO + a-PD-1: n = 5; KO + Ctrl: n = 4) were inoculated with B16-OVA (as in Fig. 2c) and treated with a-PD-1 or isotype control (Ctrl) Ab at days 3,6,9 post-implantation. Tumor volumes are shown. *Upper*, each dot represents an average of tumor volumes at a single day. *Bottom*, individual mouse at each day. WT: *Foxp3*^YFP-Cre^, KO: *Prdm1*^fl/fl^*Foxp3*^YFP-Cre^. ns, no significance, * *P* < 0.05, ** *P* < 0.01 and *** *P* < 0.001 (c-d, e (*upper*), unpaired two-tailed Student’s t-test; e (*bottom*), two-way ANOVA with Tukey’s comparisons test, Day 20: **, WT + Ctrl vs. WT + a-PD-1; *, WT + Ctrl vs. KO + Ctrl; *, WT + Ctrl vs. KO + a-PD-1. Day 21: **, WT + Ctrl vs. WT + a-PD-1; *, WT + Ctrl vs. KO + Ctrl; **, WT + Ctrl vs. KO + a-PD-1. Day 26: **, WT + Ctrl vs. WT + a-PD-1; **, WT + Ctrl vs. KO + Ctrl; ****, WT + Ctrl vs. KO + a-PD-1. Day 28: *, WT + a-PD-1 vs. KO + Ctrl; ***, WT + a-PD-1 vs. KO + a-PD-1). Bars, mean ± SEM

Finally, we determined what extent disruptions of Treg/T_FR_ suppressive activity by a specific deletion of Blimp1 could impact on the tumor by analyzing gene expression of sorted CD45^−^ cells using the NanoString PanCancer Immune Profiling Panel (Fig. 8a and Additional file 3e). Although only 38 genes were significantly differentially expressed (Additional file 9), pathway analysis revealed genes related to type 1 interferon (IFN-I) signature, including *Mx2*, *Cxcl11*, *Oas2* and *Irf7*, were enriched (*FDR < 0.05*) and downregulated, while the gene *Vegfa* encoding the angiogenic factor vascular endothelial growth factor A (VEGFA) was upregulated in CD45^−^ cells from *Prdm1*^fl/fl^*Foxp3*^YFP-Cre^ mice compared to WT mice (Fig. 8a,b and Additional file 10). Analysis using the PanCancer Pathway Panel also revealed the gene *Pgf* encoding another angiogenic factor placental growth factor (PlGF), that was significantly upregulated in CD45^−^ cells from *Prdm1*^fl/fl^*Foxp3*^YFP-Cre^ mice (Fig. 8a and Additional file 11). Accordingly, the tumor sections from *Prdm1*^fl/fl^*Foxp3*^YFP-Cre^ mice had increased numbers of CD31^+^ vessel-like structures, but with much smaller areas (Fig. 8c), suggesting a potential tumoral vasculature normalization. Further analysis showed that CD45^−^ cells from *Prdm1*^fl/fl^*Foxp3*^YFP-Cre^ mice also upregulated genes encoding MHCI and MHCII molecules as well as those related to MHCII-mediated antigen-presentation pathway, including *CD74* and *H2-DMb1* (Additional file 12a). In addition to MHCII and CD74, CD45^−^ cells from *Prdm1*^fl/fl^*Foxp3*^YFP-Cre^ mice also upregulated PD-L1, albeit no significance achieved, and had fewer Ki-67^+^ proliferating cells (Fig. 8d and Additional file 12b). All of these findings suggested that deletion of Blimp1 in Treg cells not only boosted TIL anti-tumor immune cells, but also improved tumor immunogenicity.

While IFN-I typically promotes anti-tumor immunity, persistent tumoral IFN-I signaling renders tumor resistance to checkpoint blockade therapy [40]. The increased IFNγ production in the TME of *Prdm1*^fl/fl^*Foxp3*^YFP-Cre^ mice could potentially drive prolonged IFN-I and induce adaptive resistance [40]. However, the improved tumor immunogenicity and reduced IFN-I response led us to reason that tumors from *Prdm1*^fl/fl^*Foxp3*^YFP-Cre^ mice may display an increased responsiveness to anti-PD-1 treatment. Indeed, anti-PD-1 greatly reduced tumor growth of *Prdm1*^fl/fl^*Foxp3*^YFP-Cre^ mice, while it did not significantly improve tumor control in WT mice (Fig. 8e). We also noted that PD-1 expression did not change significantly in splenic and TIL Treg cells and T_FH_ cells, as well as splenic CD8^+^ T-cells, but was increased in TIL CD8^+^ T-cells in *Prdm1*^fl/fl^*Foxp3*^YFP-Cre^ mice compared to WT mice (Additional file 12c). Further analysis of these CD8^+^ T-cells revealed that there were reduced terminally-differentiated PD-1^+^CD44^+^Tim3^+^TCF1^−^CD8^+^ T-cells but significantly increased stem-like PD-1^+^CD44^+^Tim3^−^TCF1^+^CD8^+^ T-cells [41] in the tumor of *Prdm1*^fl/fl^*Foxp3*^YFP-Cre^ mice compared to WT mice (Additional file 12d). Moreover, TIL PD1^+^CD8^+^ T-cells from *Prdm1*^fl/fl^*Foxp3*^YFP-Cre^ mice expressed increased levels of Ki-67 compared to WT counterparts (Additional file 12e). Given the recent finding that PD-1 expression balance between Teff and Treg cells can predict the clinical efficacy of PD-1 blockade therapy [42], the shifted balance of PD-1 towards TIL CD8^+^ T-cells in *Prdm1*^fl/fl^*Foxp3*^YFP-Cre^ mice may explain the favorable response of PD-1 blockade in these mice.

**Fig. 9:**
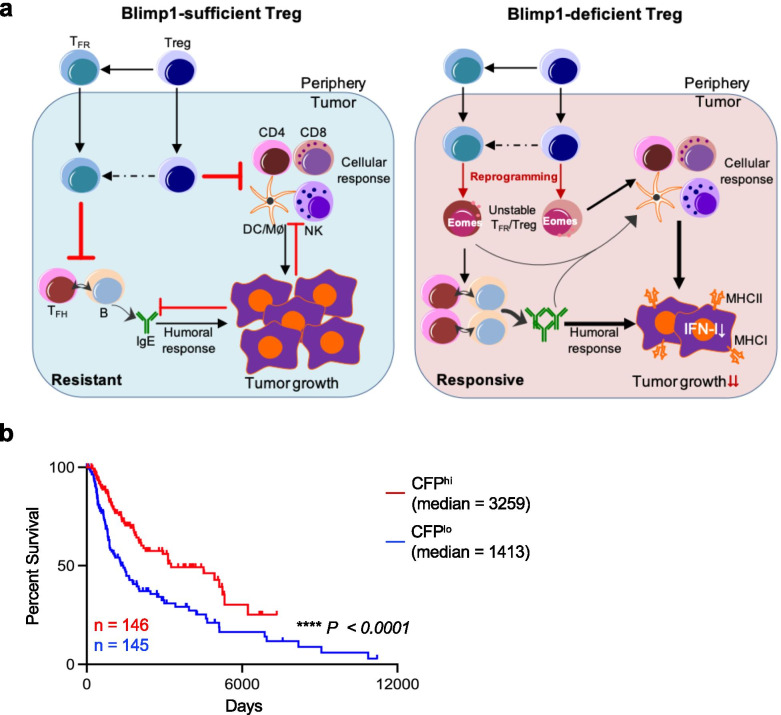
Disrupting Blimp1^+^ Treg activity reshapes the TME for improved tumor control and response to checkpoint blockade. **a**) *Left*, Blimp1-sufficient Treg. Treg and T_FR_ cells mainly suppress the cellular and humoral anti-tumor immune responses, respectively. Conversely, tumor cells impose suppression on both cellular and humoral immune responses, but foster the immune suppression by Treg and T_FR_ cells (not depicted). *Right*, Blimp1-deficient Treg. Deletion of Blimp1 in Treg cells specifically destabilizes and reprograms TIL Treg and T_FR_ cells into Teff, which upregulate Eomes, display impaired suppressive activity and cooperate with both cellular and humoral anti-tumor components to control tumor growth. Disrupting Blimp1^+^ Treg activity also increases tumor immunogenicity by upregulating MHC-related molecules, reduces IFN-I signature and augments response to checkpoint blockade therapy. MΦ: macrophage. The unclear events are indicated by dashed lines. Not depicted: peripheral T_FH_ and B-cells and their migration into the tumor; expansion of Treg/T_FR_ cells and anti-tumor effector cells; other cells regulating anti-tumor responses (e.g., myeloid-derived suppressor cells, etc.). **b**) Kaplan-Meier analysis of OS of patient cohorts expressing differential CFP signature (top 33% vs bottom 33%) based on combined log-averaging of *CD74*, *FCER1A* and *PDCD1* transcript levels from the TCGA-SKCM dataset. *P* value is generated using two-tailed LogRank test. Median, median survival time

Taken together, our results suggest that deletion of Blimp1 in Treg cells specifically converted TIL Treg and T_FR_ cells into Teff, which cooperated with both cellular and humoral anti-tumor components to reprogram the immunosuppressive TME into an immunostimulatory milieu and to enhance tumor immunogenicity, resulting in better tumor control and augmented response to anti-PD-1 blockade (Fig. 9a). This finding was further evidenced by using a quantitative measure of a putative immunostimulatory TME signature based on transcript levels of 3 factors extracted from the TCGA dataset, *CD74, FCERIA* and *PDCD1* (CFP), or levels of 20 factors comprising all HLA genes in addition to CFP (CFPHLA). These factors highly represented enhanced anti-tumor cellular and humoral immunity, particularly IgE response, in our *Prdm1*^fl/fl^*Foxp3*^YFP-Cre^ mice. The higher expression of both CFP and CFPHLA signatures was correlated with the better survival of SKCM patients (Fig. 9b and Additional file 12 f).

## Discussion

Understanding the symbiotic relationship between the tumor and TIL Treg cells is crucial for the manipulation of Treg activity for cancer therapy. The present study has revealed that Treg cells in the tumor were imprinted by the TME and regulated by Blimp1 which imposed TIL Treg cells with a unique signature responsible for their stable suppression and cytotoxicity. Deletion of Blimp1 in Treg cells reprogrammed these cells into Teff, which was specific to the TIL Treg cells but not Treg cells in the periphery, leading to increased anti-tumor cellular and humoral immunity, and decreased tumor growth. Moreover, this study has also demonstrated that remodeling the TME by disrupting Treg activity improved response to anti-PD-1 treatment.

The functional stability of Treg cells has been extensively investigated and relies on many factors under various conditions, including growing tumors [1, 3, 21, 43–46]. For example, disruption of the CARMA1–BCL10–MALT1 signalosome complex or targeting Nrp1 or Helios or ligation of GITR in Treg cells has been shown to destabilize TIL Treg cells and effectively control tumor without peripheral autoimmune effects reported [19, 21, 47–49]. Based on the finding that Blimp1 marks a subset of TIL Treg cells with the highly suppressive activity and the specific effect of Blimp1 depletion on the stable suppression of TIL Treg cells, our study has revealed Blimp1 as another central regulator of TIL Treg cells. The converted TIL Blimp1-deficient Treg cells constitute a new source of anti-tumor effector activity and targeting Blimp1^+^ Treg cells can generate robust anti-tumor effects while limiting systemic toxicity.

The mechanisms for the Blimp1-dependent regulation of stable TIL Treg cells are likely multifactorial. Under inflammation, Blimp1 can stabilize the conserved non-coding sequence 2 (*CNS2*) of *Foxp3* by either preventing its methylation or ensuring the activation of CD25-STAT5 pathway [10, 11, 13], which also operated in TIL Treg cells, as reflected by the reduced expression of *Il2ra* and pStat5 in TIL Blimp1-deficient Treg cells compared to WT Treg cells. However, other genetic or epigenetic regulation of Foxp3 expression cannot be excluded. Despite that Bcl6 antagonizes Blimp1 in many cell types [10, 27], Bcl6 was not altered in TIL Blimp1-deficient Treg cells, suggesting a Bcl6-independent role of Blimp1 in the regulation of tumor immunity. We also noted that deletion of Blimp1 reduced the TIL Treg expression of *Il10* (encoding IL-10) and *Ebi3* (encoding a subunit of IL-35). Both IL-10 and IL-35 are critical cytokines for Treg suppressive activity and important for inducing TIL CD8^+^ T-cell exhaustion [50]. Their decreased expression may partly account for the activated status of TIL CD8^+^ T-cells in *Prdm1*^fl/fl^*Foxp3*^YFP-Cre^ mice. Additionally, the gene *Tcf7* that encodes the TF TCF1 was upregulated in Blimp1-deficient Treg cells, consistent with the antagonistic regulation between TCF1 and Blimp1 [51]. Ablation of TCF1 in Treg cells blocks the development of T_FR_ cells [52], however, the frequency of TIL T_FR_ cells was not altered in mice with Blimp1-deficient Treg cells. Moreover, TCF1 partners with Foxp3 to repress the proinflammatory program in Treg cells, but not the core Treg cell transcriptional signature [53, 54]. Future investigation is required to understand the mechanisms for the overall reprogramming of Blimp1-deficient Treg cells in our system and other tumor models.

Blimp1 instructs a universal transcriptional program of tissue residency in lymphocytes [55], and Treg cells display progressive and transcriptional dynamics of adaptation to the non-lymphoid tissues, including tumor [56]. Interestingly, our RNA-seq analysis clearly showed that TIL Blimp1^+^ Treg cells developed adaptation to the TME. However, the modulation of a cytotoxic program in TIL Treg cells by Blimp1 is unexpected, although it is known that Treg cells can mediate suppression via killing. This genetic reprogramming appears to be dependent of the expression of Eomes. Although Treg cells in the periphery were not significantly altered, ablation of Eomes in TIL Blimp1-deficient Treg cells not only reduced the cytotoxic signature (e.g., IFNγ and GzmB), but also restored the stable phenotype to some degrees. Reduced IFNγ in DKO TIL Treg cells may also facilitate their stabilization, as increased IFNγ induces the Treg cell “fragility” [21]. The overall outcome may result in enhanced suppression by DKO TIL Treg cells, as reflected by reduced GzmB^+^CD8^+^ Teff, partially explaining the increased tumor growth in DKO mice compared to WT mice. It is possible that the Eomes-mediated cytotoxic program of TIL Treg cells is decoupled from the Foxp3-dependent gene signature for their stability, but both are controlled by Blimp1. Future studies are required to understand if the Eomes-dependent regulation is required for TIL Treg cells to kill tumor cells or suppress anti-tumor effector cells, and how Blimp1 regulates the TIL Treg heterogeneity.

Our study has also supported a potential role of T_FR_ cells in tumor immunity. Despite a few reports showing that T_FR_ cells are significantly increased in cancer patients compared to healthy controls [57, 58], and a recent study showing that TIL T_FR_ cells curtail anti-PD-1 therapeutic efficacy [59], their mechanisms of action in the tumor remain unclear. Consistent with the increased proportions of metastatic melanoma patients expressing high levels of *FOXP3*, *PRDM1* and *CXCR5*, we have detected T_FR_ cells in melanoma across the different metastatic tissues. Although the tissue environment may affect the Treg suppressive phenotype, the T_FR_ cell frequency was consistently and inversely correlated with the abundance of activated B-cells. Importantly, the T_FR_ adoptive transfer assay has established that dysregulated T_FR_ cells due to the deletion of Blimp1 boosted anti-tumor Ab responses, although co-transfer of other Teff, e.g., CD8^+^ T-cells, may enhance the overall tumor control. We have also shown for the first time that disruption of T_FR_ suppressive activity modulated anti-tumor immune responses, albeit no changes in T_FR_ cell numbers. It is interesting to observe that Eomes ablation in Blimp1-deficient Treg cells did not alter the T_FR_ frequency, but regulated the T_FH_-GC Ab response. Although there was no significant difference in the cellular and humoral response in mice with a Treg-specific deletion of Eomes compared to WT mice, future studies are required to understand if the changes in the T_FH_-GC Ab response in DKO mice are attributed to a direct or indirect effect of the Eomes deletion in Blimp1-deficient Treg cells. To facilitate analysis of T_FR_ cells and Ab response, we used the B16-OVA/NP-OVA model that generated strong humoral responses. However, the cellular and humoral changes in mice with the Treg-specific deletion of Blimp1 compared to WT mice were consistent across all of the models we have used. The analysis of various models has informed us that in addition to inducing Treg destabilization, targeting Blimp1^+^ Treg cells also induces potent humoral responses, thus achieving multifaceted anti-tumor effects.

Recent studies have shown that individuals with higher levels of B-cell class switches in the tumor, not only the total B-cell infiltration levels, have significantly better clinical outcomes in melanoma and other tumors [60, 61]. However, it is of interest to observe that IgE and anti-tumor specific IgE but not IgG were mainly increased in mice with a deletion of Blimp1 in Treg cells. The increased IL-4 and IL-21 produced by both T_FH_ and T_FR_ cells in *Prdm1*^fl/fl^*Foxp3*^YFP-Cre^ mice may account for the elevated IgE as we reported in other settings [10, 13], but other factors are likely involved, as revealed by recent reports [62, 63]. Consistent with these studies, our findings point to Blimp1^+^ T_FR_ cells as key suppressors of IgE production in the context of tumor, although at other settings, T_FR_ cells have been shown to induce IgE [64, 65]. Considering the IgE’s emerging anti-tumor activity [66–68], the first ongoing clinical trial using an IgE anti-tumor Ab in cancer patients (NCT02546921), the inverse correlation of serum IgE scores and risk of melanoma [32], the use of ultra-low IgE as a biomarker for cancer risk [69], and the associated usage of omalizumab, a monoclonal Ab that blocks IgE, with more cancer incidences [70], further definition of biological consequences and mechanisms of action for IgE in tumor immunity is of key importance. Although IgE may exhibit anti-tumor responses via activation of various effector cells, the preferential colocalization of IgE and its receptor FcεRIα with CD68^+^ macrophages in the tumor and increased macrophage function after treatment with sera from *Prdm1*^fl/fl^*Foxp3*^YFP-Cre^ tumor-bearing mice suggest that macrophages are likely major effector cells participating in the IgE-mediated anti-tumor response, in line with other reports [34, 35]. Notably, the finding that SKCM patients with the higher FceM1 or CFP signatures have better survival suggests that FceM1 or CFP could be used as diagnostic and prognostic markers for these patients. It is also important that a portion of IgE is specific to tumor antigen. However, we cannot exclude other portions of IgE that may exhibit autoreactive anti-tumor activity, as reported for its role in carcinogen-induced skin cancer [66]. Future delineation of the specificity and effector activity of IgE may facilitate us to understand its anti-tumor potential and any systemic reactivity.

The alterations of genes related to angiogenesis and IFN-I response in the tumor of *Prdm1*^fl/fl^*Foxp3*^YFP-Cre^ mice appear contradictory to their conventional roles in tumor control. However, the increased tumor immunogenicity along with the reduced IFN-I signature in the tumor of *Prdm1*^fl/fl^*Foxp3*^YFP-Cre^ mice, which can potentially sensitize tumors to checkpoint blockade and to potentially destabilize Treg cells [40, 71], justifies a better therapeutic outcome by combining Treg-specific deletion of Blimp1 with anti-PD-1 treatment, as proved in this study. The enhanced responsiveness to anti-PD-1 treatment may also result from the increased TIL T_FH_/GC B-cell responses, as reported by recent clinical studies [15, 16], despite no success in detecting the TLS formation in our mouse models. It should be noted that only TIL CD8^+^ T-cells but no other cells, including Treg cells, expressed increased PD-1 in *Prdm1*^fl/fl^*Foxp3*^YFP-Cre^ mice with all of the tumor models that we have evaluated. These TIL CD8^+^ T-cells displayed more of a stem-like phenotype with increased proliferation potential [41], which may contribute to the overall strong anti-tumor immunity and improved responses to anti-PD-1 blockade. Moreover, the unaltered PD-1 expression in TIL Blimp1-deficient Treg cells may suggest that PD-1 does not negatively impact these cells, as higher PD-1 levels in Treg cells impair their suppression [72, 73]. Blimp1 could act as a repressor or activator of PD-1 expression in CD8^+^ T-cells depending on the stages of immune responses [74, 75], but no definitive reports show that Blimp1 also regulates PD-1 in Treg cells, particularly TIL Treg cells, which requires further investigation.

## Conclusions

Our study has revealed that the Blimp1-dependent regulation of Treg suppression in tumor immunity extends beyond its conventional role in other settings. Although depletion or inhibition of systemic Treg cells can enhance anti-tumor responses, autoimmune sequelae have diminished the enthusiasm for such approaches. By virtue of the unique transcriptional signature of TIL Blimp1-deficient Treg cells, specific reprogramming of TIL Blimp1^+^ Treg cells and reshaping the TME are highly desirable and important for treating cancer patients, including those treated with immunotherapy, as it will direct the development of effective, targeted immunotherapies with reduced adverse events. This represents a new direction for how to manipulate Treg activity for cancer treatment and how to design combination checkpoint blockade therapies. The immune signatures that are revealed in this study as an outcome of targeted disrupting Blimp1^+^ Treg activity positively correlate with better survival of SKCM patients, suggesting the applicability of this approach for cancer therapy.

## Supplementary Information

**Additional file 1:** **Table 1.** Reagents and Resources.

**Additional file 2:** **Table 2.** Characteristics of de-identified metastatic melanoma tissues. Only patients 1-3 have matched control tissues.

**Additional file 3:** Gating strategy used for flow cytometry analysis and sorting. a-b) Gating strategy used for analysis of immune cells from spleens (a) or tumors (b) isolated from tumor-bearing mice presented on Fig. 1a-c, Fig. 2e-g, Fig. 3a-d, Fig. 4a-b,g, Fig. 7a, c-e, Fig. 8d; Additional file 4 a-b, 6, 12b-e. c) Gating strategy used for sorting of Foxp3^+^(YFP^+^)CD44^+^Treg from spleen (1→2, followed by steps 1-4 in a) and tumor (3→4, followed by steps 1-5 in b) for RNA-seq analysis presented in Fig 6 and Additional file 4 c-d. d) Gating Strategy used for analysis of immune cells from metastatic tissues of patients with melanoma presented on Fig. 1d-g. e) Gating strategy used for sorting of CD45^–^ cells for NanoString analysis presented in Fig. 8a-b and Additional file 9-11, 12a. The number at the right lower corner in each plot indicates the order of sub-gating for each condition.

**Additional file 4: **Blimp1^+^ Treg cells are accumulated in the tumor. a-b) Blimp1-YFP reporter mice (*n* = 5) were inoculated with B16-OVA and immunized as in Fig. 1a. Flow plots of CD62L^lo^CD44^hi^Foxp3^+^eTreg, Blimp1^+^(YFP^+^) eTreg and IL-10^+^Blimp1^+^Treg subset (a) as quantitated in Fig. 1a, and MFI of each marker of Blimp1^+^Foxp3^+^Treg cells (b) as presented in Fig. 1b. c-d) *Foxp3*^YFP-Cre^ mice were established with B16-OVA/NP-OVA model as in Fig. 1a. eTreg cells (CD45^+^CD44^+^YFP^+^CD4^+^CD3^+^) from spleens or tumors were sorted for RNA-seq (duplicates). Principle component analysis of splenic and TIL eTreg cells (c), top KEGG pathways that are differentially expressed in splenic versus TIL eTreg cells (analyzed by g:Gost) (d). * *P* < 0.05, ** *P* < 0.01 and *** *P* < 0.001 (b, unpaired two-tailed Student’s t-test). Bars, mean ± SEM. e-g) The correlation of *PRDM1* and *FOXP3* expression in all SKCM patients (*n* = 458) (e) or the correlation of *PRDM1* and *FUT4* expression (f) or *PRDM1* and *FUT7* expression (g) in top 50% *FOXP3*^*hi*^ SKCM patients (*n* =229) (extracted from the TCGA dataset) was analyzed by Pearson correlation (two-tailed, no adjustment for multiple comparisons because of one correlation test for a gene pair). The values of the coefficients (r) and significance (p) are indicated.

**Additional file 5: Table 3.** Quantitation of each marker or subset in metastatic melanoma tissues compared to control tissues. *P value: unpaired two-tailed Student’s t-test. Numbers indicate mean ± SEM. ns, no significance.

**Additional file 6: **TIL effector cells and expression of effector molecules in TIL or splenic effector cells and Treg cells. B16-OVA/NP-OVA model was established in *Foxp3*^YFP-Cre^ (WT) and *Prdm1*^fl/fl^*Foxp3*^YFP-Cre^ (KO) mice, as in Fig. 2c. a) Frequency of TIL immune cells (*n* = 7 per group, except *n* = 3 (WT) and *n* = 4 (KO) for F4/80^+^ cells). b-c) Frequency of each effector subset in spleens (WT: *n* = 8; KO: *n* = 6) (b) or tumors (n = 7 per group) (c) expressing IFNγ, TNFα and GzmB. d) Analysis and frequency of splenic Treg cells expressing IFNγ, TNFα and GzmB (WT: n = 8; KO: n = 6). ns, no significance, *** *P* < 0.001 and **** *P* < 0.0001 (a-d, unpaired two-tailed Student’s t-test). Bars, mean ± SEM.

**Additional file 7: **Immunofluorescence (IF) staining of CD3, B220 and IgE in the spleens of *Prdm1*^fl/fl^*Foxp3*^YFP-Cre^ mice, as positive controls for Fig. 4F. Spleens were taken from mice bearing B16-OVA (as in Fig. 2c). Representative IF staining of T (CD3) and B (B220) (a), or B220 and IgE (b, *left*) or CD3, IgE and IgD (b, *right*) (100 ×). IgE^+^ cells are localized outside germinal centers. Arrowheads, T/B clusters; *, IgE.

**Additional file 8: **Comparison and quantitation of pStat5 (a) and pSmad2/3 (b) levels in TIL Treg cells from *Foxp3*^YFP-Cre^ (WT) and *Prdm1*^fl/fl^*Foxp3*^YFP-Cre^ (KO) mice (n = 3 per group) established with B16-OVA, as in Fig. 2c. ∆MFI: MFI subtracted from the MFI of isotype controls (Ctrl). ns, no significance and * *P* < 0.05 (unpaired two-tailed Student’s t-test). Bars, mean ± SEM.

**Additional file 9:** **Table 4.** NanoString mouse PanCancer Immune Profiling of sorted CD45^−^ cells (KO (n =2) vs WT (n =3)).

**Additional file 10:** **Table 5.** Reactome pathway analysis of DEGs revealed in Table 4 (KO (n =2) vs WT (n =3)) via NetworkAnalyst.

**Additional file 11:** **Table 6.** NanoString mouse PanCancer Pathway analysis of sorted CD45^−^ cells (KO vs WT, n = 2 per group).

**Additional file 12: **MHC and PD-L1 expression in CD45^−^ cells and PD-1 expression in each immune subset. B16-OVA/NP-OVA model was established in *Foxp3*^YFP-Cre^ (WT) and *Prdm1*^fl/fl^*Foxp3*^YFP-Cre^ (KO) mice, as in Fig. 2c. a) DEGs related to MHCI and MHCII in WT (n = 3) and KO mice (n = 2), as revealed by NanoString analysis in Fig. 8a. b) PD-L1 MFI in CD45^−^ cells (WT: n = 3; KO: n = 4). c) PD-1 MFI in each subset (WT: *n* = 9; KO: n = 8). d) Flow plots of Tim3 and TCF-1 expression in PD-1^+^CD44^+^CD8^+^ T-cells in the tumor from B16-OVA mice (WT: n = 3; KO: n = 4), as in Fig. 2c. *Right*, frequency of indicated CD8^+^ T-cell subsets. e) Comparison and quantitation of Ki-67 in TIL PD-1^+^ CD8^+^ T-cells from B16-OVA mice (WT: n = 3; KO: n = 4), as in Fig. 2c. Data represent one of two (b,d,e) or are pooled from two (c) independent experiments. ns, no significance and * *P* < 0.05 (unpaired two-tailed Student’s t-test). Bars, mean ± SEM. f) Kaplan-Meier analysis of OS of patient cohorts expressing differential CFPHLA signature (top 33% vs bottom 33%) based on combined log-averaging of transcript levels of 20 genes (*right*) from the TCGA-SKCM dataset. *P* value is generated using two-tailed LogRank test. Median, median survival time.

## Data Availability

The RNA sequencing and NanoString data have been deposited in the NCBI GEO under accession number GSE178135. All data generated or analyzed during this study are included in this article [and its supplementary information (Additional files)].

## Abbreviations

Ab: Antibody
ACK: Ammonium-Chlorine-Potassium
BMDMs: Bone marrow-derived macrophages
CFA: Complete Freund’s adjuvant
CNS2: Conserved non-coding sequence 2
CTV: Cell trace violet
DC: Dendritic cell
DEGs: Differentially expressed genes
DKO: Double knockout
DMEM: Dulbecco’s Modified Eagle Medium
ELISA: Enzyme-Linked Immunosorbent Assay
Eomes: Eomesodermin
eTreg: Effector regulatory T-cell
FACS: Flow activated cell sorting
FBS: Fetal bovine serum
FFPE: Formalin-fixed paraffin embedded
GC: Germinal center
GM-CSF: Granulocyte-macrophage colony stimulating factor
GzmB: Granzyme B
GVAX: B16 expressing GM-CSF
IFN-I: Type I interferon
IF: Immunofluorescence
IFA: Incomplete Freund’s adjuvant
IL: Interleukin
i.p.: Intraperitoneally
MFI: Mean fluorescence intensity
MHCI or MHCII: Major histocompatibility complex class I or class II
NP-OVA: 4-Hydroxy-3-nitrophenylacetyl-Ovalbumin
OCT: Optimum cutting temperature
OVA: Ovalbumin
OS: Overall survival
pAcc: Probability of accumulation
PBS: Phosphate-buffered saline
PlGF: Placental growth factor
pORA: Probability of over-representation
RSEM: RNA-Seq by Expectation-Maximization
RT: Room temperature
s.c.: Subcutaneously
SEM: Standard error of the mean
SKCM: Skin Cutaneous Melanoma
TCGA: The Cancer Genome Atlas
TCR: T cell receptor
Teff: Effector T-cells
TF: Transcription factor
T_FH_: Follicular helper T cells
T_FR_: Follicular regulatory T cells
TLS: Tertiary lymphoid structure
TME: Tumor microenvironment
TPM: Transcripts Per Million
Treg: Regulatory T-cell
VEGFA: Vascular endothelial growth factor A
