# Supplementary material for: Remodeling of the tumor microenvironment via disrupting Blimp1+ effector Treg activity augments response to anti-PD-1 blockade
Source: Mol Cancer. 2021 Nov 20;20:150. doi: 10.1186/s12943-021-01450-3 (PMC8605582; doi:10.1186/s12943-021-01450-3)
Supplement: Supplementary file 1 — Additional file 1: Table 1. Reagents and Resources. [file 12943_2021_1450_MOESM1_ESM.docx]

**Additional file 1.** Table 1: Reagents and Resources

| REAGENT or RESOURCE | SOURCE | CATALOGUE |
| --- | --- | --- |
| Antibodies/Dilution | | |
| Purified Anti-Mouse CD16/CD32 (Fc Block)/ (1:100) | Biolegend | Cat# 101320 |
| Anti-mouse CD45 (30-F11)/ (1:300); (1:250) | Biolegend | Cat# 103151; 103124 |
| Anti-mouse CD4 (GK1.5)/ (1:250) | BD Biosciences | Cat# 552051 |
| Anti-mouse CD4 (RM4-5)/ (1:250); (1:250) | Biolegend | Cat# 100531; 100559 |
| Anti-mouse CD3 (145-2C11)/ (1:250); (1:250) | Biolegend | Cat# 100233; 100306 |
| Anti-mouse CD3 (145-2C11)/ (1:250) | BD Biosciences | Cat# 612771 |
| Anti-mouse CD8a (53-6.7)/ (1:250) | Biolegend | Cat# 100744 |
| Anti-mouse NKp46 (29A1.4)/ (1:250) | Biolegend | Cat# 137621 |
| Anti-mouse CD25 (PC61)/ (1:300) | BD Biosciences | Cat# 553866 |
| Anti-mouse CD25 (PC61)/ (1:250) | Biolegend | Cat# 102025 |
| Anti-mouse GITR (DTA-1)/ (1:250); (1:400) | Biolegend | Cat# 126308; 126317 |
| Anti-mouse CTLA4 (UC10-4B9)/ (1:100); (1:50) | Biolegend | Cat# 106306; 106311 |
| Anti-mouse Blimp1 (5E7)/ (1:50) | BD Biosciences | Cat# 563643 |
| Anti-mouse Granzyme B (NGZB)/ (1:50) | eBioscience | Cat# 11889882 |
| Anti-mouse Granzyme B (GB11)/ (1:50) | Biolegend | Cat# 515408 |
| Anti-mouse Helios (22F6) / (1:50); (1:50) | Biolegend | Cat# 137229; 137220 |
| Anti-mouse CD19 (1D3)/ (1:250) | BD Biosciences | Cat# 551001 |
| Anti-mouse CD19 (B4)/ (1:250) | Biolegend | Cat# 115543 |
| Anti-mouse CD44 (IM7)/ (1:250) | Biolegend | Cat# 103028 |
| Anti-mouse CD62L (MEL-14)/ (1:250) | Biolegend | Cat# 104441 |
| Anti-mouse Fas (15A7)/ (1:250) | BD Biosciences | Cat# 554258 |
| Anti-mouse T- and B-cell activation antigen (GL7)/ (1:250); (1:250) | Biolegend | Cat# 144606; 144612 |
| Anti-mouse PD-1 (J43)/ (1:300) | BD Biosciences | Cat# 551892 |
| Anti-mouse PD-1 (29F.1A12)/ (1:400); (1:250) | Biolegend | Cat# 135216; 135220 |
| Anti-mouse IFN-γ (XMG1.2)/ (1:50) | BD Biosciences | Cat# 554412 |
| Anti-mouse IL-10 (JES5-16E3)/ (1:50) | BD Biosciences | Cat# 554467 |
| Anti-Human/mouse Bcl6 (K112-91)/ (1:50) | BD Biosciences | Cat# 561522 |
| Anti-mouse FoxP3 (FJK-16s)/ (1:150); (1:100) | eBioscience | Cat# 25577382; 45577382 |
| Anti-mouse CXCR5 (2G8)/ (1:150) | BD Biosciences | Cat# 551960 |
| Anti-mouse CD38 (90)/ (1:250) | Biolegend | Cat# 102732 |
| Streptavidin-APC/ (1:250) | Biolegend | Cat# 405207 |
| Streptavidin-APC.Cy7/ (1:200) | Biolegend | Cat# 405208 |
| Anti-mouse Gr-1 (RB6-8C5)/ (1:250) | Biolegend | Cat# 108430 |
| Anti-mouse/human CD11b (M1/70)/ (1:250) | Biolegend | Cat# 101245 |
| Anti-mouse CD11c (N418)/ (1:400) | Biolegend | Cat# 117317 |
| Anti-mouse I-A/I-E (M5/114.15.2)/ (1:1000) | Biolegend | Cat# 107639 |
| Anti-mouse F4/80 (BM8)/ (1:250) | Biolegend | Cat# 123129 |
| Anti-mouse CD206 (C068C2)/ (1:250) | Biolegend | Cat# 141727 |
| Anti-mouse TNF-α (MP6-XT22)/ (1:200) | Biolegend | Cat# 506322 |
| Anti-mouse IgE (RME-1)/ (1:200) | Biolegend | Cat# 406909 |
| Anti-mouse Eomes (Dan11mag)/ (1:200); (1:200) | eBioscience | Cat# 46487582; 50487580 |
| Anti-mouse CD74 (In1/CD74)/ (1:250) | Biolegend | Cat# 151004 |
| Anti-mouse PD-L1 (10F.9G2)/ (1:250) | Biolegend | Cat# 124315 |
| Anti-mouse Ki-67 (16A8)/ (1:400) | Biolegend | Cat# 652420 |
| Anti-mouse Tim3/ (1:400) | Biolegend | Cat# 119715 |
| Anti-human/mouse TCF1/ (1:50) | BD Biosciences | Cat# 564217 |
| Anti-mouse Phospho-STAT5 (Tyr694)/ (1:200) | Cell Signaling Technology | Cat# 9351 |
| pSMAD2/3/ (1:50) | BD Biosciences | Cat# 562586 |
| Purified goat anti-mouse IgG/ (1:960) | Jackson ImmunoResearch | Cat# 115-005-008 |
| Goat anti-mouse IgG HRP/ (1:1000) | Invitrogen | Cat# A16084 |
| Purified NA/LE anti-mouse CD3 (145-2C11)/ 5 μg/ml | BD Biosciences | Cat# 553057 |
| Purified NA/LE anti-mouse CD28 (37.51)/ 2 μg/ml | BD Biosciences | Cat# 553294 |
| Anti-human CD16/CD32/ (1:100) | Biolegend | Cat# 422302 |
| Anti-human CD3 (UCHT1)/ (1:250) | Biolegend | Cat# 300463 |
| Anti-human CD4 (RPA-T4)/ (1:250) | Biolegend | Cat# 300545 |
| Anti-human CD25 (M-A251)/ (1:250) | Biolegend | Cat# 356111 |
| Anti-human CD127 (A019D5)/ (1:250) | Biolegend | Cat# 351325 |
| Anti-human CD19 (SJ25C1)/ (1:250) | Biolegend | Cat# 363015 |
| Anti-human IgD (IA6-2)/ (1:250) | Biolegend | Cat# 348227 |
| Anti-human Blimp1 (646702)/ (1:50) | RD Systems | Cat# IC36081G |
| Anti-human IL-10 (JES3-19F1)/ (1:50) | Biolegend | Cat# 506804 |
| Anti-human CTLA4 (BNI3)/ (1:50) | Biolegend | Cat# 369624 |
| Anti-human FoxP3 (259D)/ (1:50) | Biolegend | Cat# 320215 |
| Anti-human Helios (22F6)/ (1:50) | eBioscience | Cat# 56988341 |
| Anti-human CXCR5 (Poly4053)/ (1:250) | Biolegend | Cat# 405322 |
| Anti-human Bcl6 (7D1)/ (1:50) | Biolegend | Cat# 358509 |
| Anti-human PD-1 (EH12.2H7)/ (1:400) | Biolegend | Cat# 329918 |
| Anti-human CD38 (HB7)/ (1:250) | BD Biosciences | Cat# 612825 |
| Anti-human CD27 (O323)/ (1:300) | Biolegend | Cat# 302842 |
| Alexa Fluor 594 rat anti-mouse CD45R/B220 (RA3-6B2)/ (1:250) | Biolegend | Cat# 103254 |
| Alexa Fluor 488 rat anti-mouse CD45R/B220 (RA3-6B2)/ (1:250) | Biolegend | Cat# 103225 |
| Alexa Fluor 647 rat anti-mouse CD31 (390)/ (1:250) | Biolegend | Cat# 102415 |
| Alexa Fluor 488 rat anti-mouse CD68 (FA-11)/ (1:250) | Biolegend | Cat# 137011 |
| Purified rat anti-mouse IgE (R35-92)/ (1:250) | BD Biosciences | Cat# 553416 |
| Purified Armenian Hamster anti-mouse FcεRIα (MAR-1)/ (1:250) | Biolegend | Cat# 134336 |
| Alexa Fluor 647 rat anti-mouse IgD (11-26c.2a)/ (1:500) | Biolegend | Cat# 405708 |
| Human CXCR5 Antibody (51505)/ (1:20) | RD Systems | Cat# MAB190-100 |
| Rat FOXP3 Monoclonal Antibody (PCH101)/ (1:50) | eBioscience | Cat# 14477682 |
| Rabbit CD4 Monoclonal Antibody (SP35)/ (1:50) | Millipore Sigma | Cat# 104R-14 |
| Alexa Fluor 488 donkey anti-rabbit IgG/ (1:200) | Biolegend | Cat# 406416 |
| Alexa Fluor 555 goat anti-rat IgG/ (1:500) | Biolegend | Cat# 405420 |
| Alexa Fluor 647 goat anti-mouse IgG/ (1:200) | Biolegend | Cat# 405322 |
| Alexa Fluor 594 Goat anti-hamster (Armenian) IgG Antibody (poly4055)/ (1:300) | Biolegend | Cat# 405512 |
| Anti-mouse PD-1 (RMP1-14)/ 200 µg/dose | BioXcell | Cat# BE0146 |
| Rat IgG2a isotype Control (2A3)/ 200 µg/dose | BioXcell | Cat# BE0089 |
| Chemicals, Peptides, and Recombinant Proteins | | |
| ACK lysing buffer | Millipore Sigma | Cat# R7757 |
| G418 | Fisher Scientific | Cat# 10131035 |
| Collagenase/Dispase | Millipore Sigma | Cat# 11097113001 |
| DNase I | Millipore Sigma | Cat# 10104159001 |
| Ficoll-Paque 1.084 | Fisher Scientific | Cat# 45001755 |
| Tamoxifen | Millipore Sigma | Cat# T5648 |
| Sunflower seed Oil | Millipore Sigma | Cat# S5007 |
| CFA | Millipore Sigma | Cat# F5881 |
| IFA | Millipore Sigma | Cat# F5506 |
| NP_16_-OVA (16 loading) | Biosearch Technologies | Cat# N-5051-100 |
| OVA | InvivoGen | Cat# vac-stova |
| M-CSF | Biolegend | Cat# 576402 |
| Leukocyte Activation cocktail | BD Biosciences | Cat# 550583 |
| O.C.T Compound | Fisher Scientific | Cat# 23730571 |
| IHC Antigen Retrieval Solution (high pH) | eBioscience | Cat# 00495658 |
| ProLong Diamond Antifade Mountant with DAPI | Invitrogen | Cat# P36962 |
| Critical Commercial Assays | | |
| FoxP3 staining Buffer Set | eBioscience | Cat# 00552300 |
| Fixable Viability Dye | eBioscience | Cat# 65086314 |
| LIVE/DEAD Fixable Near-IR Dead Cell Stain Kit | Invitrogen | Cat# L34976 |
| Cell Trace Violet Proliferation Kit | Life Technologies | Cat# C34571 |
| IgE OptEIA ELISA Set | BD Biosciences | Cat# 555248 |
| RNeasy plus micro kit | Qiagen | Cat# 74034 |
| Mouse CD4 microbeads | Miltenyi Biotec | Cat# 130049201 |
| Mouse CD4+ CD25+ Regulatory T Cell Isolation Kit | Miltenyi Biotec | Cat# 130091041 |
| Mouse CD8a (Ly-2) microbeads | Miltenyi Biotec | Cat# 130117044 |
| Deposited Data | | |
| RNAseq dataset | This paper | GEO: GSE178135 |
| NanoString Data | This paper | GEO: GSE178135 |
| Experimental Models: Cell Lines | | |
| B16-F10 | ATCC | Cat# CRL-6475 |
| B16-OVA | Nakagawa et al., 2016 | N/A |
| B16-GMCSF | Nakagawa et al., 2016 | N/A |
| MC38 | Nakagawa et al., 2016 | N/A |
| Experimental Models: Organisms/Strains | | |
| Mouse: B6: C57BL/6J | Jackson Laboratories | Jax:000664 |
| Mouse: *Prdm1*^fl/fl^: B6.129-*Prdm1*^tm1Clme^/J | Jackson Laboratories | Jax:008100 |
| Mouse: *FoxP3^YFP-Cre^*: B6.129(Cg)-*Foxp3^tm4(YFP/cre)Ayr^/J* | Jackson Laboratories | Jax:016959 |
| Mouse: B6.129-*Gt(ROSA)26Sor*^tm1(cre/ERT2)Tyj^/J | Jackson Laboratories | Jax:008463 |
| Mouse: *Tcrα^–/–^:* B6.129S2-*Tcra*^tm1Mom^/J | Jackson Laboratories | Jax:002116 |
| Mouse: Blimp1-YFP: B6.Cg-Tg(Prdm1-EYFP)1Mnz/J | Jackson Laboratories | Jax:008828 |
| Mouse: *Eomes*^fl/fl^*:* B6.129S1(Cg)-*Eomes*^tm1.1Bflu^/J | Jackson Laboratories | Jax:017293 |
| Software and Algorithms | | |
| FlowJo | FlowJo, LLC | v. 10.6 |
| FACSDiva | BD Bioscience | v. 8 |
| Prism 8 | GraphPad | v. 8 |
| OpenLAB software | Agilent Technologies | v. 3.1 |
| ImageJ | NIH | v. 1.50i |
| Oncolnc | N/A | <http://www.oncolnc.org> |
| iPathawayGuide | Advaita Bioinformatics | <https://ipathwayguide.advaitabio.com> |
| g:Gost | g:Profiler | https://biit.cs.ut.ee/gprofiler/gost |
| Trimmomatic | N/A | v.0.36 |
| STAR aligner | N/A | v.2.5.2b |
| Subread package (featureCounts) | N/A | v.1.5.2 |
| DESeq2 | N/A | DOI: 10.18129/B9.bioc.DESeq2 |
| nSolver | NanoString Technologies | v.4.0 |
| NetworkAnalyst | N/A | https://www.networkanalyst.ca |
