# Supplementary figures and images for: Remodeling of the tumor microenvironment via disrupting Blimp1+ effector Treg activity augments response to anti-PD-1 blockade
Source: Mol Cancer. 2021 Nov 20;20:150. doi: 10.1186/s12943-021-01450-3 (PMC8605582; doi:10.1186/s12943-021-01450-3)

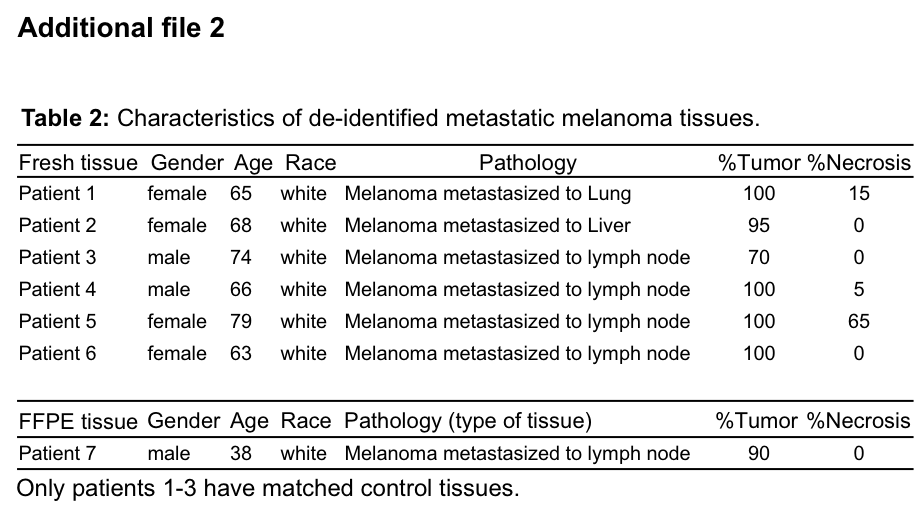

Supplement: Supplementary file 2 — Additional file 2: Table 2. Characteristics of de-identified metastatic melanoma tissues. Only patients 1-3 have matched control tissues. [file 12943_2021_1450_MOESM2_ESM.docx]

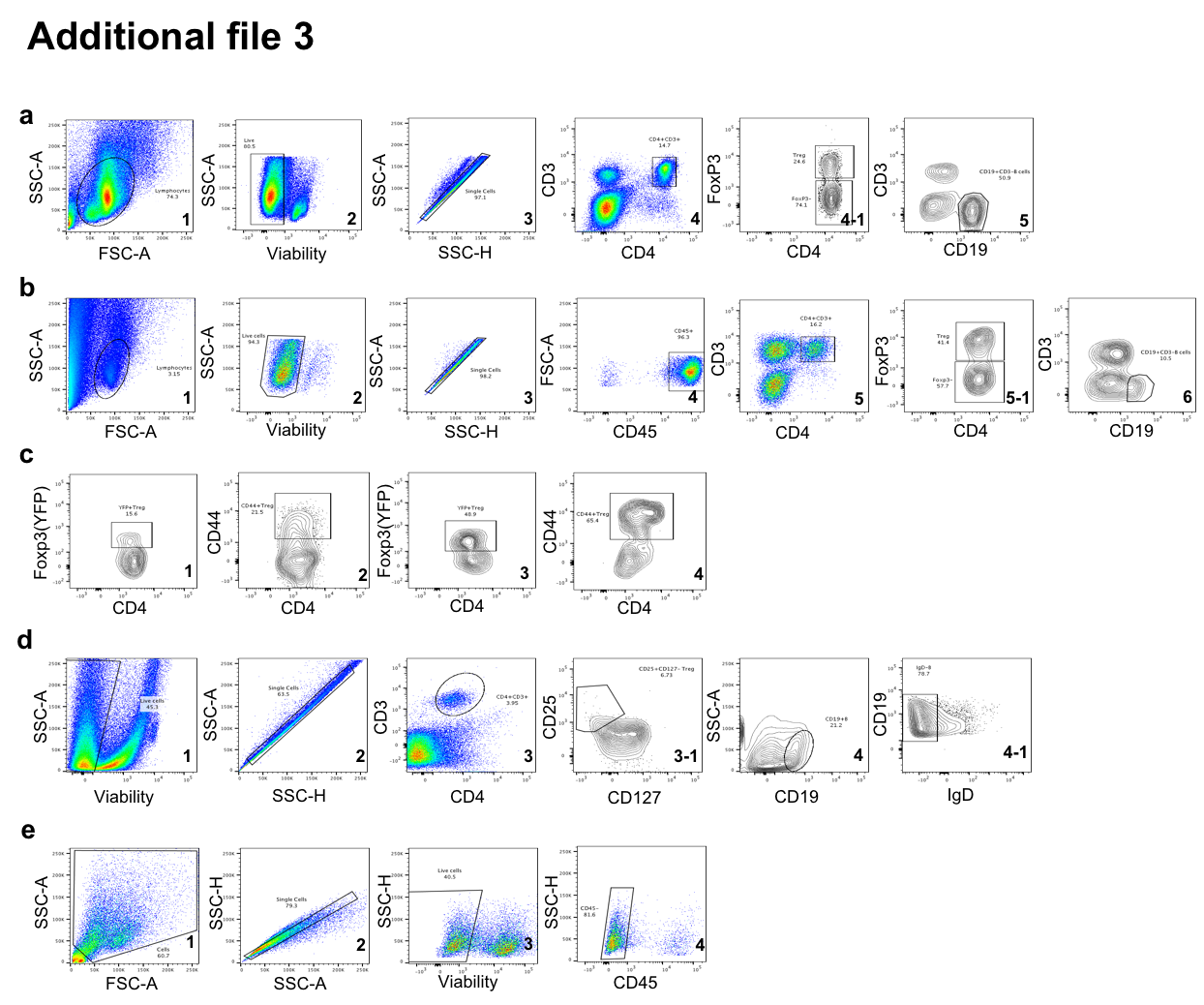

Supplement: Supplementary file 3 — Additional file 3: Gating strategy used for flow cytometry analysis and sorting. a-b) Gating strategy used for analysis of immune cells from spleens (a) or tumors (b) isolated from tumor-bearing mice presented on Fig. 1a-c, Fig. 2e-g, Fig. 3a-d, Fig. 4a-b,g, Fig. 7a, c-e, Fig. 8d; Additional file 4 a-b, 6, 12b-e. c) Gating strategy used for sorting of Foxp3+(YFP+)CD44+Treg from spleen (1→2, followed by steps 1-4 in a) and tumor (3→4, followed by steps 1-5 in b) for RNA-seq analysis presented in Fig 6 and Additional file 4 c-d. d) Gating Strategy used for analysis of immune cells from metastatic tissues of patients with melanoma presented on Fig. 1d-g. e) Gating strategy used for sorting of CD45– cells for NanoString analysis presented in Fig. 8a-b and Additional file 9-11, 12a. The number at the right lower corner in each plot indicates the order of sub-gating for each condition. [file 12943_2021_1450_MOESM3_ESM.docx]

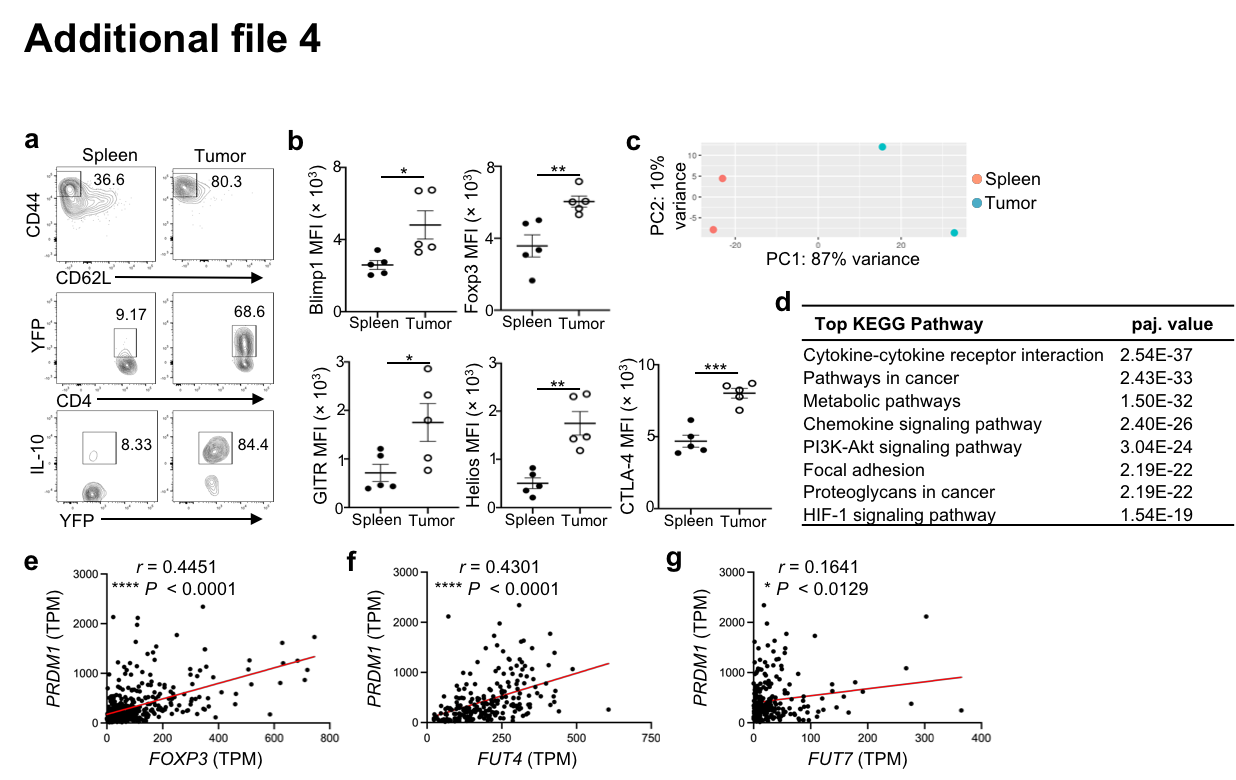

Supplement: Supplementary file 4 — Additional file 4: Blimp1+ Treg cells are accumulated in the tumor. a-b) Blimp1-YFP reporter mice (n = 5) were inoculated with B16-OVA and immunized as in Fig. 1a. Flow plots of CD62LloCD44hiFoxp3+eTreg, Blimp1+(YFP+) eTreg and IL-10+Blimp1+Treg subset (a) as quantitated in Fig. 1a, and MFI of each marker of Blimp1+Foxp3+Treg cells (b) as presented in Fig. 1b. c-d) Foxp3YFP-Cre mice were established with B16-OVA/NP-OVA model as in Fig. 1a. eTreg cells (CD45+CD44+YFP+CD4+CD3+) from spleens or tumors were sorted for RNA-seq (duplicates). Principle component analysis of splenic and TIL eTreg cells (c), top KEGG pathways that are differentially expressed in splenic versus TIL eTreg cells (analyzed by g:Gost) (d). * P < 0.05, ** P < 0.01 and *** P < 0.001 (b, unpaired two-tailed Student’s t-test). Bars, mean ± SEM. e-g) The correlation of PRDM1 and FOXP3 expression in all SKCM patients (n = 458) (e) or the correlation of PRDM1 and FUT4 expression (f) or PRDM1 and FUT7 expression (g) in top 50% FOXP3hi SKCM patients (n =229) (extracted from the TCGA dataset) was analyzed by Pearson correlation (two-tailed, no adjustment for multiple comparisons because of one correlation test for a gene pair). The values of the coefficients (r) and significance (p) are indicated. [file 12943_2021_1450_MOESM4_ESM.docx]

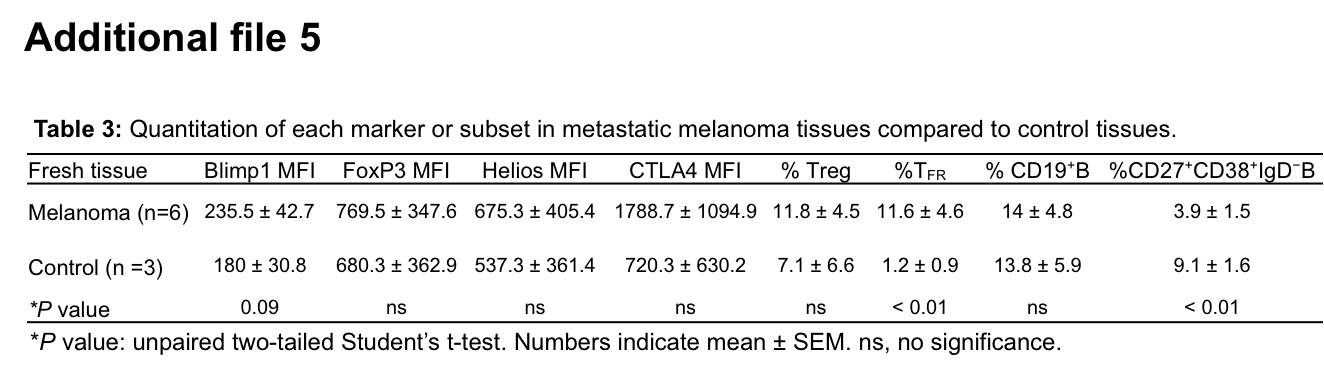

Supplement: Supplementary file 5 — Additional file 5: Table 3. Quantitation of each marker or subset in metastatic melanoma tissues compared to control tissues. *P value: unpaired two-tailed Student’s t-test. Numbers indicate mean ± SEM. ns, no significance. [file 12943_2021_1450_MOESM5_ESM.docx]

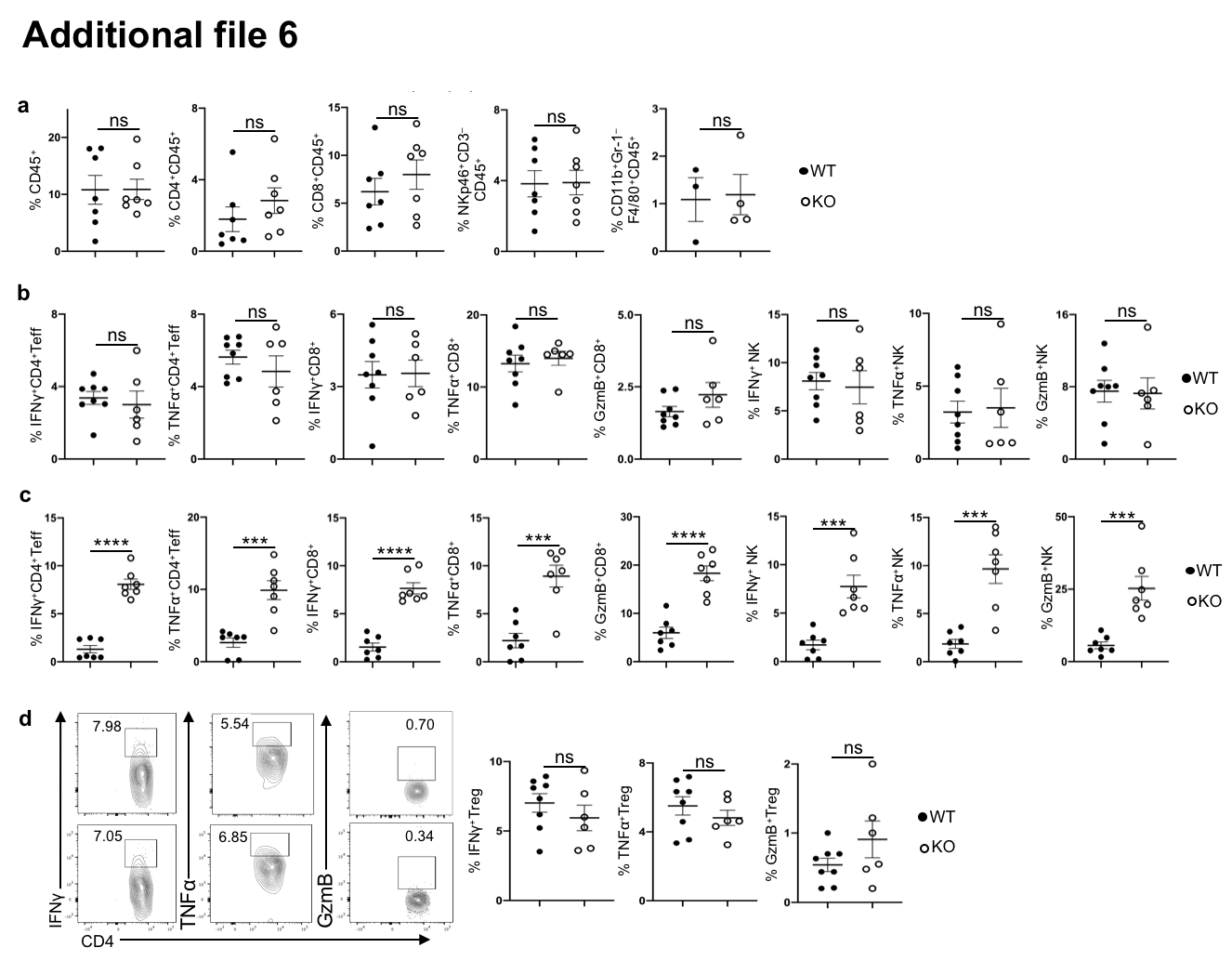

Supplement: Supplementary file 6 — Additional file 6: TIL effector cells and expression of effector molecules in TIL or splenic effector cells and Treg cells. B16-OVA/NP-OVA model was established in Foxp3YFP-Cre (WT) and Prdm1fl/flFoxp3YFP-Cre (KO) mice, as in Fig. 2c. a) Frequency of TIL immune cells (n = 7 per group, except n = 3 (WT) and n = 4 (KO) for F4/80+ cells). b-c) Frequency of each effector subset in spleens (WT: n = 8; KO: n = 6) (b) or tumors (n = 7 per group) (c) expressing IFNγ, TNFα and GzmB. d) Analysis and frequency of splenic Treg cells expressing IFNγ, TNFα and GzmB (WT: n = 8; KO: n = 6). ns, no significance, *** P < 0.001 and **** P < 0.0001 (a-d, unpaired two-tailed Student’s t-test). Bars, mean ± SEM. [file 12943_2021_1450_MOESM6_ESM.docx]

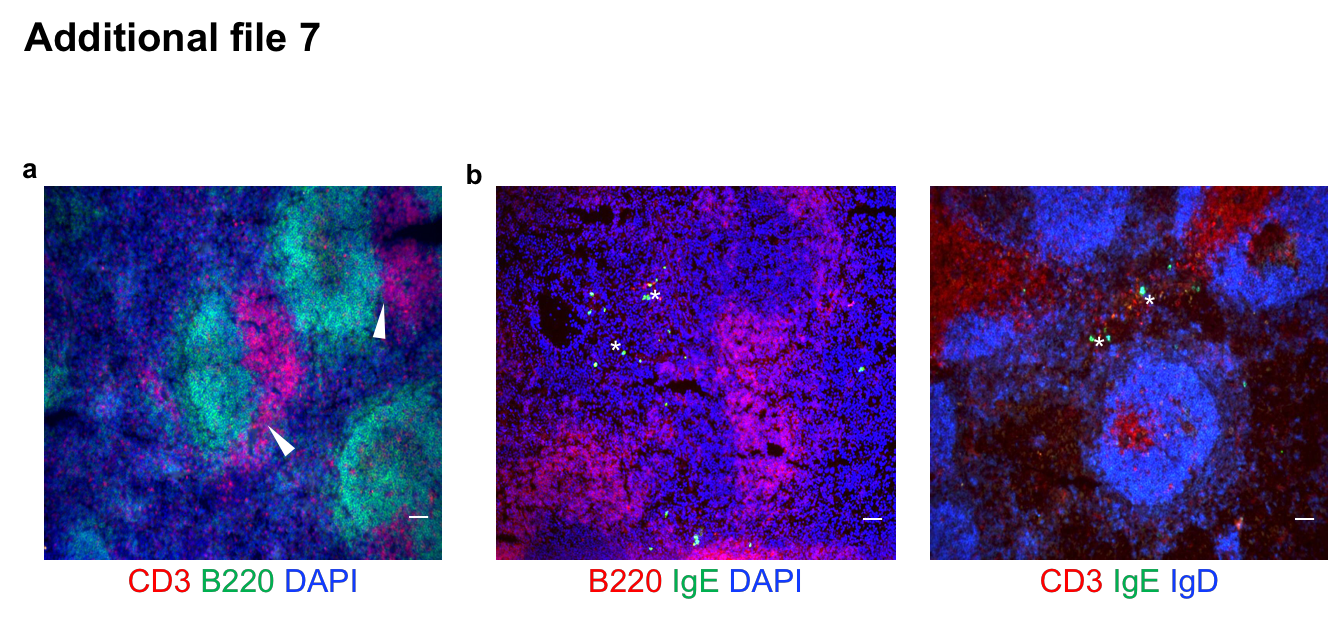

Supplement: Supplementary file 7 — Additional file 7: Immunofluorescence (IF) staining of CD3, B220 and IgE in the spleens of Prdm1fl/flFoxp3YFP-Cre mice, as positive controls for Fig. 4F. Spleens were taken from mice bearing B16-OVA (as in Fig. 2c). Representative IF staining of T (CD3) and B (B220) (a), or B220 and IgE (b, left) or CD3, IgE and IgD (b, right) (100 ×). IgE+ cells are localized outside germinal centers. Arrowheads, T/B clusters; *, IgE. [file 12943_2021_1450_MOESM7_ESM.docx]

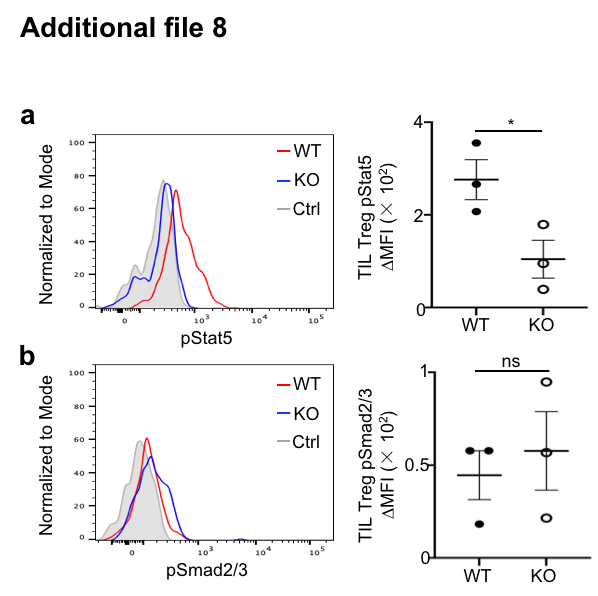

Supplement: Supplementary file 8 — Additional file 8: Comparison and quantitation of pStat5 (a) and pSmad2/3 (b) levels in TIL Treg cells from Foxp3YFP-Cre (WT) and Prdm1fl/flFoxp3YFP-Cre (KO) mice (n = 3 per group) established with B16-OVA, as in Fig. 2c. ∆MFI: MFI subtracted from the MFI of isotype controls (Ctrl). ns, no significance and * P < 0.05 (unpaired two-tailed Student’s t-test). Bars, mean ± SEM. [file 12943_2021_1450_MOESM8_ESM.docx]

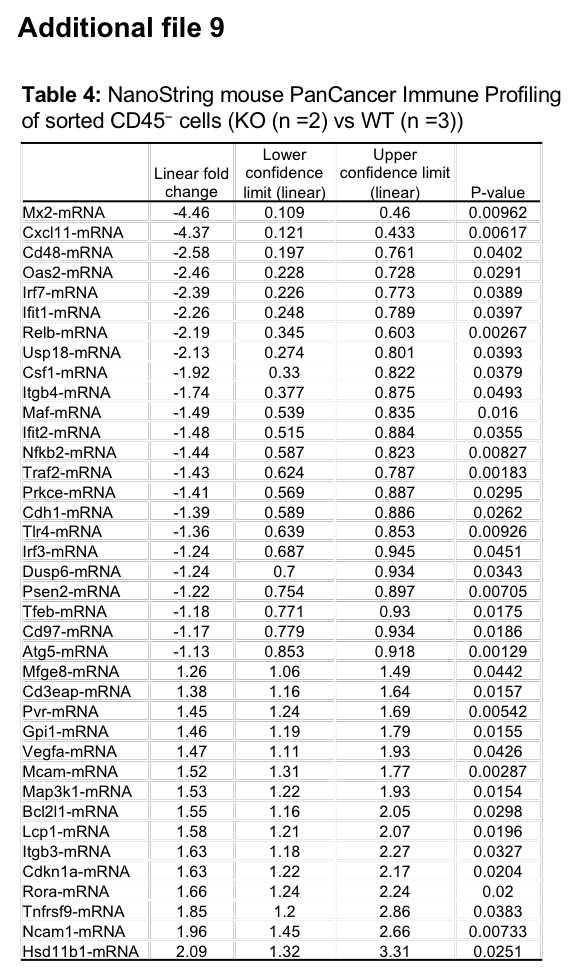

Supplement: Supplementary file 9 — Additional file 9: Table 4. NanoString mouse PanCancer Immune Profiling of sorted CD45− cells (KO (n =2) vs WT (n =3)). [file 12943_2021_1450_MOESM9_ESM.docx]

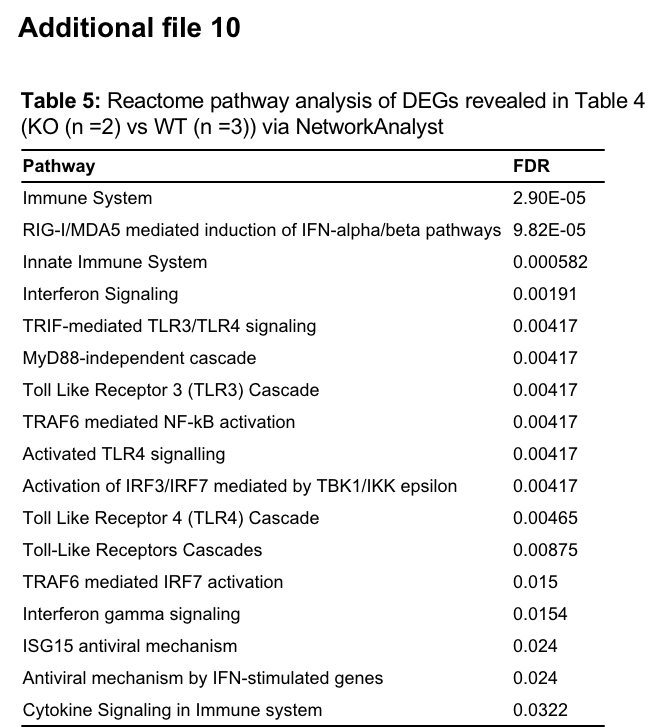

Supplement: Supplementary file 10 — Additional file 10: Table 5. Reactome pathway analysis of DEGs revealed in Table 4 (KO (n =2) vs WT (n =3)) via NetworkAnalyst. [file 12943_2021_1450_MOESM10_ESM.docx]

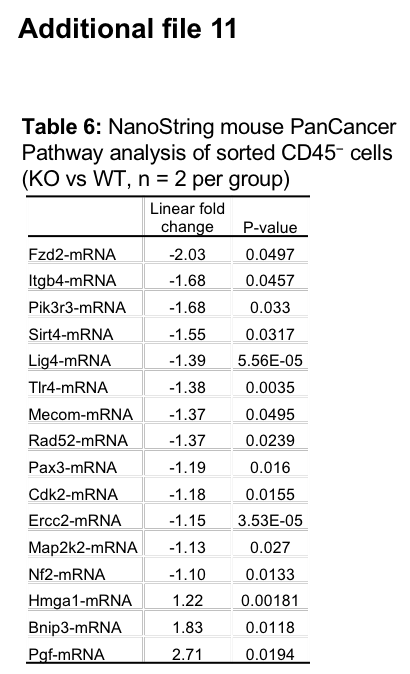

Supplement: Supplementary file 11 — Additional file 11: Table 6. NanoString mouse PanCancer Pathway analysis of sorted CD45− cells (KO vs WT, n = 2 per group). [file 12943_2021_1450_MOESM11_ESM.docx]

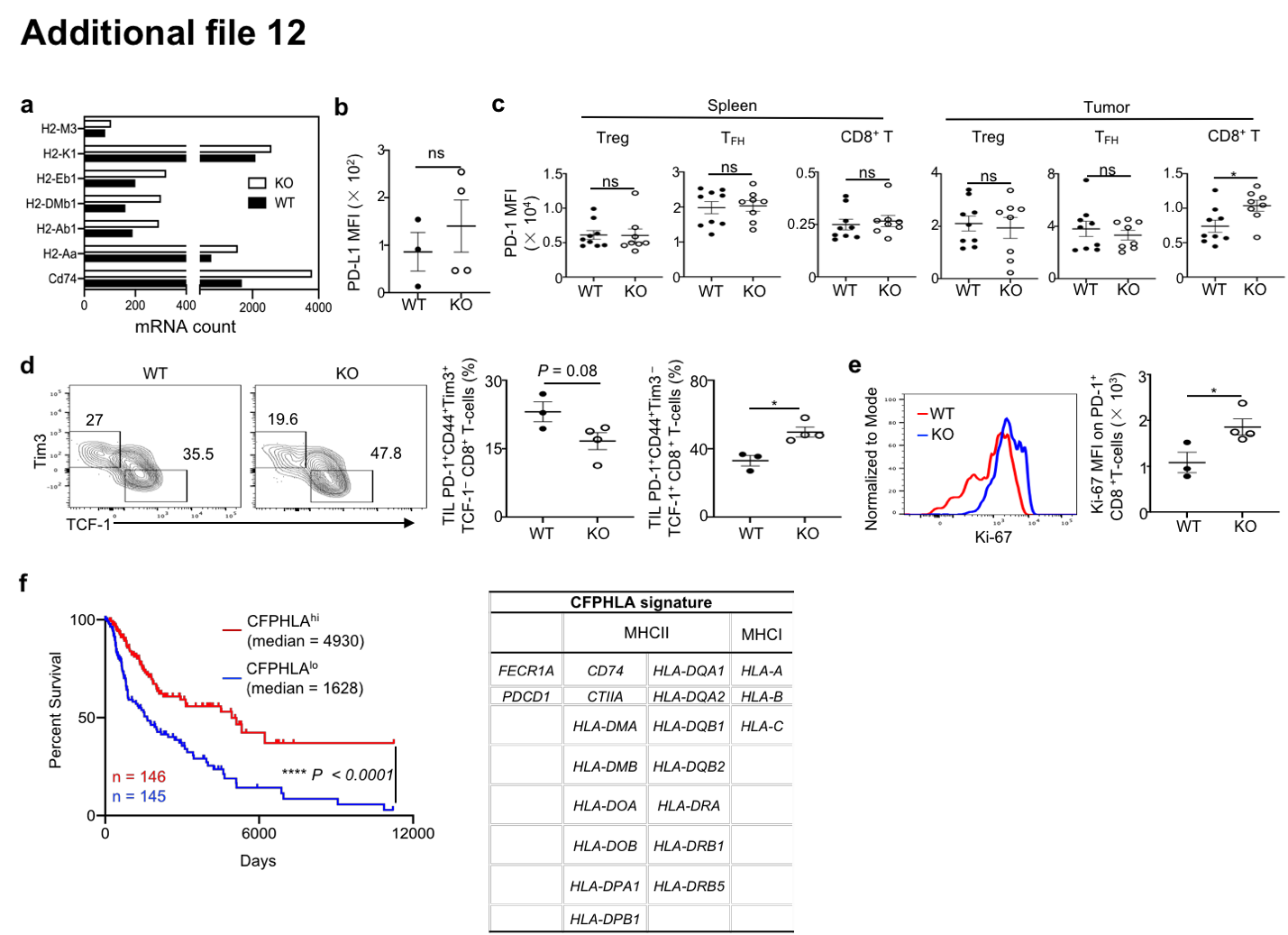

Supplement: Supplementary file 12 — Additional file 12: MHC and PD-L1 expression in CD45− cells and PD-1 expression in each immune subset. B16-OVA/NP-OVA model was established in Foxp3YFP-Cre (WT) and Prdm1fl/flFoxp3YFP-Cre (KO) mice, as in Fig. 2c. a) DEGs related to MHCI and MHCII in WT (n = 3) and KO mice (n = 2), as revealed by NanoString analysis in Fig. 8a. b) PD-L1 MFI in CD45− cells (WT: n = 3; KO: n = 4). c) PD-1 MFI in each subset (WT: n = 9; KO: n = 8). d) Flow plots of Tim3 and TCF-1 expression in PD-1+CD44+CD8+ T-cells in the tumor from B16-OVA mice (WT: n = 3; KO: n = 4), as in Fig. 2c. Right, frequency of indicated CD8+ T-cell subsets. e) Comparison and quantitation of Ki-67 in TIL PD-1+ CD8+ T-cells from B16-OVA mice (WT: n = 3; KO: n = 4), as in Fig. 2c. Data represent one of two (b,d,e) or are pooled from two (c) independent experiments. ns, no significance and * P < 0.05 (unpaired two-tailed Student’s t-test). Bars, mean ± SEM. f) Kaplan-Meier analysis of OS of patient cohorts expressing differential CFPHLA signature (top 33% vs bottom 33%) based on combined log-averaging of transcript levels of 20 genes (right) from the TCGA-SKCM dataset. P value is generated using two-tailed LogRank test. Median, median survival time. [file 12943_2021_1450_MOESM12_ESM.docx]
